# LC-MS/MS Fingerprinting Analysis of *Cyanotis arachnoidea* Extracts: Process-Related Artifacts in Anabolic Food Supplements

**DOI:** 10.1021/acsomega.4c10908

**Published:** 2025-05-02

**Authors:** Dávid Laczkó, En-Liang Chu, Ching-Chia Chang, Fang-Rong Chang, Gábor Girst, Tamás Gáti, Gábor Tóth, Árpád Könczöl, Attila Hunyadi

**Affiliations:** † Institute of Pharmacognosy, University of Szeged, Szeged H-6720, Hungary; ‡ HUN-REN-SZTE Biologically Active Natural Products Research Group, Szeged H-6720, Hungary; § Graduate Institute of Natural Products, Kaohsiung Medical University, Kaohsiung 80780, Taiwan; ∥ RotaChrom Technologies LLC, Kecskemét 6000, Csillag u. 2a, Hungary; ⊥ Botany Division, Endemic Species Research Institute, Nantou 552, Taiwan; # Servier Research Institute of Medicinal Chemistry (SRIMC), Budapest H-1031, Hungary; ∇ NMR Group, Department of Inorganic and Analytical Chemistry, Budapest University of Technology and Economics, Budapest H-1111, Hungary

## Abstract

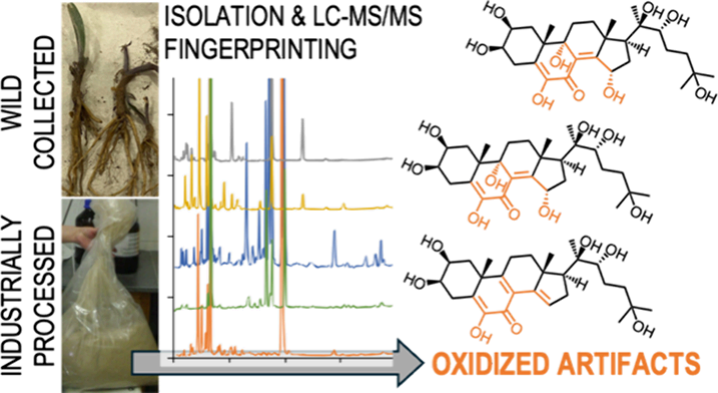

We report the isolation and complete NMR characterization of four new ecdysteroids and LC-MS/MS fingerprinting of ecdysteroids in two native and two industrially processed *Cyanotis arachnoidea* extracts along with an autoxidized product mixture of 20-hydroxyecdysone (20E). The autoxidation of 20-hydroxyecdysone leads to the formation of various unknown or uncharacterized ecdysteroid compounds, which can significantly impact the quality and efficacy of commercial ecdysteroid-containing supplements, potentially affecting their regulatory status and consumer safety. A total of 146 ecdysteroids were detected. Among these, 20 were identified using authentic and fully characterized reference standards, including the newly reported compounds. The autoxidative origin of many process-related artifacts was confirmed in the two commercial ecdysteroid extracts. Considering the pharmacological versatility of ecdysteroids and the major pharmacological differences between these compounds and their autoxidized derivatives, our results are of importance regarding the efficacy and safety of commercially available ecdysteroid-containing food supplements.

## 1 Introduction

High-resolution analytical techniques, such as “Chemical Fingerprint Analysis”, are gaining more and more attention in the characterization and quantification of complex plant extracts. This is especially true for medicinal plants and botanical supplements intended for human consumption. The goal of fingerprint analysis of plant materials can be multilateral: standardization,^1^ quality control,^2^ inspection of authenticity,^3^ metabolomic analysis,^4^ taxonomical evaluation,^5^ and interpretation of the way of processing (e.g., identifying extraction-related artifacts)^6^ are typical scenarios. Targeted and untargeted LC-MS and NMR methods in combination with chemometrics (e.g., principal component analysis) are the most powerful approaches to date.^3^

Ecdysteroids are well known as analogues of the insect molting hormone 20-hydroxyecdysone. These structurally diverse compounds play a key role in arthropod development and molting-related physiological and biochemical processes.^7^ In addition to this, ecdysteroids are present in massive quantities in a wide range of plant species; therefore, they cannot be clearly classified into animal or plant categories.^8^ The protein synthesis-enhancing effects of the compounds have been observed in insects but also in vertebrates, where an increase in body weight gain has been observed.^9^ The significant anabolic activity of these compounds has not escaped the attention of athletes, and in 2020, the World Anti-Doping Agency (WADA) added the most abundant ecdysteroid, 20-hydroxyecdysone (ecdysterone; 20E), to its monitoring program as an anabolic agent.^10^ Most recently, we have reported the ability of 20E and one of its oxidized derivative, calonysterone, to prevent high-sugar-high-fat diet (HFHSD)-induced obesity and metabolic syndrome in rats.^11^ There is a huge demand for ecdysteroid-containing dietary supplements that can be easily seen by a simple search on the Internet. There are numerous companies that offer tons of 20E or 20E-containing extracts, sometimes with a minimum order limit of hundreds of kilograms.^12^ These extracts are usually made from roots of a plant cultivated in China, *Cyanotis arachnoidea* C. B. Clarke (Commelinaceae), whose ecdysteroid content can reach as high as 3–4%, while plants containing dietary ecdysteroid such as spinach contain much lower amounts (0.005–0.08%).^13^ In addition to their anabolic potential, ecdysteroids exert several other bioactivities in mammals, including adaptogenic,^14^ antidiabetic,^15^ and neuroprotective effect;^16^ furthermore, their semisynthetic derivatives exert antimicrobial^17^ and/or chemosensitizing activity.^18^ It is of particular importance that 20E was revealed as a life-saving agent against respiratory failure in severe COVID-19 in a Phase 2/3 clinical study.^19^ It must be stressed that ecdysteroids represent a very high chemical diversity; 578 naturally occurring analogues have been reported to date, and their known variability makes over 1000 natural derivatives possible. This chemical diversity clearly represents a versatile pharmacology. Even though 20E is relatively well studied, even at the clinical level, almost nothing is known about the bioactivity and safety profile of the minor accompanying ecdysteroids.

Many studies have addressed the issue of proper analytical separation of ecdysteroid mixtures, which is an ongoing challenge due to the structural diversity and complex samples. Reversed-phase HPLC-MS techniques are preferentially used to resolve more complex samples.^12^ Using RP-HPLC-MS/MS techniques, mixtures of up to 20 elements can be analyzed with high confidence;^20^ however, as the ecdysteroid content of samples can vary from simple to very complex, fingerprint analysis can provide a solution for such samples with up to 50 components.^21^ Several coupling techniques have been attempted in the past to provide more accurate analysis.^22−24^ With the chance of 20E becoming a doping-controlled substance, increasing attention is given to the quantitative analysis of this compound and its metabolites in human biological samples.^25−27^

To this end, most if not all related research has been focusing on 20E and hardly anything is known about the ecdysteroid composition of herbal food supplements that are consumed by people. Uncharacterized bioactive compounds may potentially lead to unknown deviation from the targeted bioactivity profile, and adverse reactions or unwanted side effects may as well arise, i.e., the potency may vary unmeasurably by consumers. With these in mind, the objective of our study was (i) to optimize a high-resolution LC-MS/MS method capable of in-depth characterization of native and industrially processed ecdysteroid-containing plant extracts by detecting up to 100 components and (ii) to use this method for a comparative analysis to search for potential artifacts with pharmacological relevance.

## 2 Materials and Methods

### 2.1 Chemicals and Standards

The organic solvents and additives used for method development and LC-MS were purchased from Molar Chemicals Ltd. (Halásztelek, Hungary). The ecdysteroid compounds used as analytical standards had a purity of ≥97% by HPLC and were isolated in our previous phytochemical studies.^28,29^

### 2.2 Raw Materials

The crude extract (CAPR1) was previously prepared as published before.^30^ A commercially available *Cyanotis arachnoidea* root extract, claimed to contain 50% of 20E by means of UV absorbance, was purchased from Xi’an Olin Biological Technology Co., Ltd. (Xi’an, People’s Republic of China). This extract was percolated with methanol at room temperature and then evaporated to dryness. The other crude extract (CAPR2), claimed to contain 10.85% of 20E, was purchased from Kingherbs Limited (People’s Republic of China). Authentic *C. arachnoidea* whole plants were collected in Taiwan, separated into roots and herbs, and extracted with methanol to obtain CARO and CALF extracts, respectively. The auto-oxidated 20E sample was prepared as follows: 3 g of 20E was dissolved in 32 mL of methanol, and then 112 mL of water was added to the solution. Separately, 2,4 g of NaOH was dissolved in 24 mL of water, and then the two solutions were mixed and stirred for 6 h at room temperature to convert 20E. Subsequently, HCl was added to create an acidic environment, and the mixture was stirred at room temperature overnight. After neutralization of the reaction mixture using a NaOH solution, evaporation was carried out under reduced pressure at 40 °C. The base-catalyzed autoxidation of 20E leads to several specific structural alterations. Because oxidation by oxygen dissolved in the solvent is initiated by a 6-enolate ion, this process primarily affects the steroid skeleton and somewhat less the side chain. Largely depending on the pH and time of the reaction, a highly complex mixture of derivatives may be formed. Several new, conjugated double bonds and hydroxyl groups are typically formed on the B-, C-, and D-rings, 3-dehydro derivatives may be formed, and 5- and/or 14-epimerization, dehydration reactions, and ring expansion may also occur.^28,31^

### 2.3 Analytical Methods and Instrumentation

The HR-MS analysis of the compounds was carried out on an Agilent 1100 LC-MS instrument (Agilent Technologies, Santa Clara, California, USA) coupled with a Thermo Q-Exactive Plus orbitrap spectrometer (Thermo Fisher Scientific, Waltham, Massachusetts, USA) used in positive ionization mode. Regarding the samples, 100 μg/mL solutions were prepared with acetonitrile solvent containing 0.1% formic acid.

^1^H (600 and 500 MHz) and ^13^C (150 and 125 MHz) NMR spectra were recorded at room temperature on Bruker Avance III HD NMR spectrometers equipped with Prodigy and cryo probeheads, using MeOH-*d*_4_ or DMSO-*d*_6_ as the solvents. Chemical shifts (δ) are given on a δ scale and referenced to the solvents (CH_3_OH-*d*_4_: δH = 3.31 and δC = 49.1 ppm, and DMSO-*d*_6_: δH = 2.50 and δC = 39.5 ppm). Approximately 1–5 mg of compounds was measured in 2.5 mm Bruker MATCH NMR sample tubes or in 5 mm NMR sample tubes. Pulse programs for all experiments, i.e., ^1^H and ^13^C NMR, DEPTQ, DEPT135, APT, 1D selTOCSY, 1D selROE (τmix: 300 ms), 2D ^1^H,^1^H–COSY, HSQC, edHSQC, and HMBC (optimized for 8 Hz), were taken from the Bruker software library (TopSpin 3.5). For 1D measurements, 64,000 data points were used to obtain the FID. For 2D measurements, 2000 × 256 or 1000 × 128 data points (t2 × t1) were generally acquired, respectively. For F1, a linear prediction was applied to enhance resolution.

An RP-HPLC-MS method was developed and used for the analytical resolution of the samples. Analysis was performed on an Agilent 1260 Infinity II instrument coupled to an Agilent 6420 QQQ-ESI-MS instrument (Agilent Technologies, Santa Clara, California). A Kinetex F5, 150 × 4.6 mm; 2.6 μm column was used with a flow rate of 0.9 mL/min. The solvents used were, respectively, A: 95 v/v% 5 mM ammonium formate, 0.1 v/v% formic acid + 5 v/v% ACN and B: 95 v/v% ACN + 5 v/v% 5 mM ammonium formate, 0.1 v/v% formic acid. The gradient was the following: 0–8 min 13 to 20 v/v% B, 8–12 min 20 v/v% B, 12–24 min 20 to 40 v/v% B, and then 24–26 min 40 to 90 v/v% B. The column temperature was set to 40 °C, and the UV detection was carried out at wavelengths of 210 ± 4, 248 ± 4, and 360 ± 4 nm. LC/ESI-MS was carried out in the positive ion mode from *m*/*z* 200–800, the heated capillary temperature was set to 300 °C, and the electrospray voltage was at 4 kV, respectively. The mass spectrometer was used in data independent acquisition mode (DIA) for the analysis of crude samples, and data independent mode (DDA) composed of two scan events for the proper analysis of the reference compounds. The full-scan mass spectrum was first obtained, followed by collision-induced dissociation of the selected abundant ion from the full scan (see the Supporting Information: Table S2 for the exact CE values of measured compounds).

## 3 Results and Discussion

**Figure 1:**
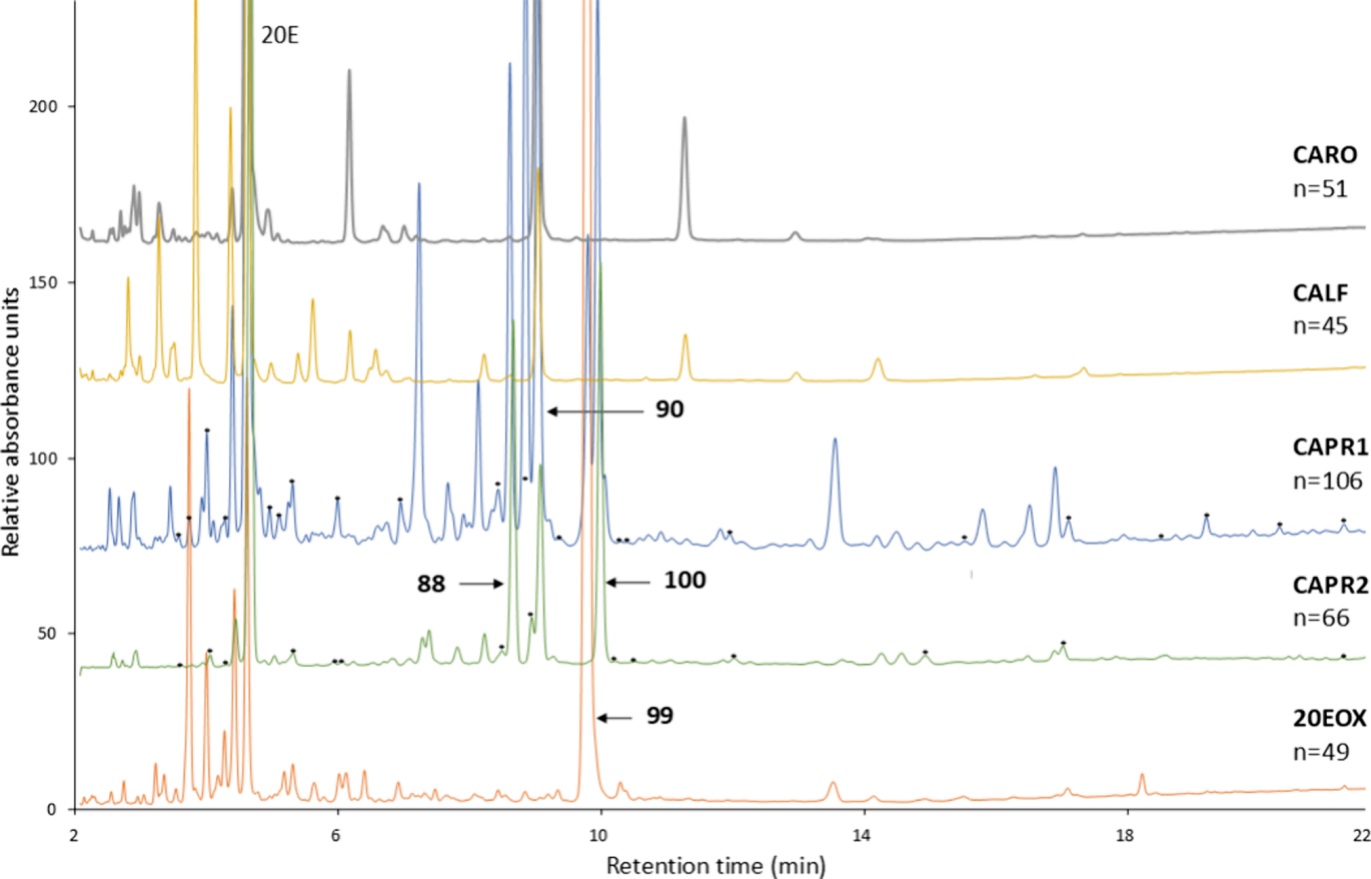
Representative RP-HPLC chromatograms of the analyzed samples recorded at 248 ± 4 nm and a number of ecdysteroid-like compounds. Compounds identified only in commercial extracts and absent from authentic natural extracts are marked with asterisks (*) on the chromatograms of CAPR1 and CAPR2. Some major ecdysteroid compounds are indicated, respectively.

Five ecdysteroid-containing samples were included in our study. First, two independent commercial extracts of *Cyanotis arachnoidea* (CAPR1 and 2) were purchased from the People’s Republic of China. Second, an authentic sample of *C. arachnoidea* was collected in Taiwan and separated into roots and leaves that were extracted with methanol; hereinafter, the extracts are termed CARO and CALF, respectively. During preliminary analysis, it was observed that the CAPR1 extract contains a much larger number of components than the authentic extracts, which may suggest artifact formation during industrial processing. To elaborate on this hypothesis, 20E base-catalyzed autoxidation was also performed and the product mixture (20EOX) served as our fifth analyte. One of the most widely used high-performance chromatographic techniques, reversed-phase HPLC coupled with tandem mass spectrometry, was chosen for the analysis of the ecdysteroid fingerprints. A Kinetex F5 column with a superficially porous pentafluorophenyl propyl stationary phase was used for the separation that is an alternative to conventional C18 columns with similar retention but orthogonal selectivity. The length of the HPLC separation was designed as a compromise between a fast run, which would result in many coeluting components and reduced mass spectrometric information, and a long run, which would result in significantly broadened peaks. Representative chromatograms of the five samples are shown in Figure 1.

The reference standards came in part from our previous phytochemical studies. As a continuation of this work, CAPR1 was also further processed by an excessive multistep combined preparative chromatographic procedure (for a detailed description, see the Supporting Information). This resulted in the isolation of four new ecdysteroids (**79**, **111**, **131**, and **136**) and one ecdysteroid (**135**) previously published with partial NMR signal assignment. The characteristic HR-MS and NMR spectra of compounds **79**, **111**, **131**, **136**, and **135** are presented as the Supporting Information (Methods S1), and to facilitate the understanding of the ^1^H and ^13^C signal assignments, the stereostructures are also depicted in the spectra. Structure elucidation was performed using comprehensive one- and two-dimensional NMR methods using widely accepted strategies,^32,33^ based on which we established the complete ^1^H and ^13^C signal assignment of the compounds. Most ^1^H assignments were achieved using a general knowledge of chemical shift dispersion with the aid of the *J*(H,H) coupling pattern (^1^H NMR spectra).

### Compound **79**

HR-MS data (Supporting Information, Figure S1) indicated an elemental composition of C_27_H_42_O_6_. For structure elucidation and NMR signal assignments (see Table 1), the following NMR spectra were recorded: ^1^H NMR; ^13^C NMR and DEPT135; edHSQC; HMBC+Me section; ^1^H,^1^H–COSY; sel-ROE on H_3_-19 and H_3_-18 and edHSQC+sel-ROE on H_3_-18 (Supporting Information, Figures S2–S8). The ^1^H and ^13^C spectra verified the presence of five methyl groups, seven CH_2_ groups, three CH and three O–CH methines, two quaternary carbon atoms, and two oxygenated quaternary carbon atoms. In the sp^2^=C area, the characteristic signals of the O=C–C=CH–C=CH (6-one-Δ^7,8;14,15^-diene) chromophore were detected. Using the selROE experiments, the assignment of the α/β configuration of the CH_2_ atoms was achieved, and the ^1^H,^1^H–COSY and HMBC cross peaks of the HO groups also enabled their assignments.

**Table 1:** ^1^H and ^13^C NMR Spectroscopic Data for Compounds **79**, **111**, **131**, **136**, and **135**

		**79**^a^		**111**^b^		**131**^b^		**136**^b^		**135**^a^	
no.		^1^H	^13^C	^1^H	^13^C	^1^H	^13^C	^1^H	^13^C	^1^H	^13^C
1	α	1.42	42.2	1.64	41.1	2.23	38.4	2.18	34.0	1.30	36.3
	β	1.91		2.16		1.70		1.83		1.70	
2	α	3.76	68.4	5.12	73.4	3.86	67.2	4.84	73.2	1.63	30.6
	β									1.18	
3		3.39	70.8	3.70	71.0	5.05	71.7	3.98	66.1	3.37	68.7
4	α	1.72	24.1	2.05	25.8	1.62	33.3	1.63	35.8	1.92	30.2
	β	1.49		1.72		1.84		1.74		1.16	
5		2.21	53.8	2.44	54.9	2.30	52.3	2.50	51.6	2.17	52.8
6			198.8		201.7		206.0		206.3		199.6
7		6.01	121.2	6.12	122.4	5.77	119.1	5.77	119.3	5.68	121.6
8			154.0		157.4		156.7		156.7		164.6
9		2.24	50.5	2.36	52.6		135.7		135.5	2.64	45.4
10			38.2		39.7		40.9		41.2		37.9
11	α	1.71	20.12	1.79	21.8	6.32	134.4	6.41	134.5	1.64	20.1
	β	1.57		1.72						1.47	
12	α	1.44	39.0	1.44	40.8	2.73	39.2	2.75	39.2	1.48	30.7
	β	2.14		2.26		2.43		2.43		1.66	
13			47.0		48.9		48.0		48.0		46.6
14			148.8		150.6		84.5		84.6		82.7
15	α	6.02	128.2	6.06	130.1	1.81	31.5	1.81	31.5	1.51	30.3
	β					1.97		1.96		1.77	
16	α	2.10	30.5	2.25	31.9	1.79	21.8	1.78	21.9	1.51	20.2
	β	2.51		2.60		2.01		2.03		1.86	
17		2.00	57.2	2.16	59.0	2.48	50.6	2.48	50.6	2.21	48.7
18		1.02	19.5	1.13	20.1	0.90	18.2	0.91	18.2	0.76	17.1
19		0.89	14.9	0.96	15.4	1.14	31.6	1.13	31.4	0.72	12.6
20			75.0		77.2		77.7		77.7		75.6
21		1.10	20.11	1.24	20.4	1.19	20.9	1.19	20.9	1.04	20.9
22		3.13	76.5	3.32	78.6	3.34	78.1	3.34	78.1	3.13	75.5
23		1.45	26.0	1.60	27.3	1.57	30.6	1.58	30.6	1.37	29.1
		1.12		1.30		1.24		1.24		1.07	
24		1.65	41.4	1.80	42.2	1.49	37.7	1.48	37.7	1.37	36.2
		1.25		1.43		1.24		1.24		1.14	
25			68.8		71.4	1.58	29.3	1.58	29.3	1.50	27.5
26		1.04	29.0	1.18	28.9	0.92	22.9	0.92	22.8	0.85	22.3
27		1.07	30.0	1.21	30.1	0.93	23.5	0.93	23.5	0.86	23.1
2-OH		4.15									
3-OH		4.54								4.60	
14-OH										4.64	
22-OH		3.73								3.58	
23-OH		4.41								4.37	
25-OH		4.12									
2-OAc				2.07	21.4			2.07	21.2		
					172.7				172.5		
3-OAc						2.12	21.3				
							172.7				

a Measured in DMSO-d_6_.

b Measured in methanol-d_4_.

### Compound **111**

HR-MS data (Supporting Information, Figure S9) established a molecular formula of C_29_H_44_O_7_. Structure elucidation and NMR assignments (see Table 1) were based on the following spectra: ^1^H NMR; ^13^C NMR and DEPT135; selROE on CH_3_-18; ^13^C NMR and DEPT135; edHSQC; edHSQC CH_2_ section; HMBC+Me section; ^1^H,^1^H–COSY; selROE on H_3_-19 + selTOCSY on H-15 and H-3 (Supporting Information Figures S10–S16). The ^1^H and ^13^C data of this compound are rather like those of **80**, with the difference that instead of an HO–, there is now an −OAc group (2.07s 3H; 21.4 and 172.7 ppm), which is also confirmed by the molecular formula. The detected vicinal *J*(H,H) coupling and the considerable diamagnetic shifts on δH-2 (5.12q ppm, ^3^*J* ∼ 3.5 Hz) and δC-2 (73.4 ppm) revealed the position C-2 of the −OAc group and the equatorial, Hα-2 configuration.

### Compound **131**

HR-MS data (Supporting Information, Figure S17) established a molecular formula of C_29_H_44_O_7_. Structure elucidation and NMR assignments (see Table 1) were based on the following spectra: ^1^H NMR; ^13^C DEPTQ; HSQC; edHSQC CH_2_ section; HMBC+Me section; selROE on H_3_-18, H_3_-19, and H_3_-21; selTOCSY on H-2 and H-11 (Supporting Information, Figures S18–24). The detected NMR data were rather similar to those published for dacryhainansterone (**112**),^34^ but the MS and ^1^H and ^13^C signals at 2.12s 3H, 21.3 and 172.7 ppm in this compound revealed the presence of an −OAc group. Compared to that of 134, strong deshielding of HC-3 signals (δH-3 5.05q ^3^*J* ∼ 3.5 Hz and δC-2 71.7 ppm) revealed the C-3 position of the −OAc group. In the A-ring, some signals, e.g., δH_3_-19 = 1.14, δC-19 = 31.6, and δC-4 = 33.3 ppm, appeared broadened, and the latter was barely detectable in the DEPTQ spectrum (Supporting Information, Figure S19). To overcome this problem, we utilized the selTOCSY experiment on H-2, which allowed a nonoverlapping measurement of the A-ring hydrogen signals (Supporting Information, Figure S24). The edHSQC cross peaks (Figure S21) of 4β = 1.84 and 4α = 1.62 ppm assigned δC-4 = 33.3 ppm. The near-coalescence state may indicate that the steric interaction of the axial 3-OAc group in the A ring may cause a special, partially hindered conformational interconversion.

### Compound **136**

HR-MS data (Supporting Information, Figure S25) established a molecular formula of the C_29_H_44_O_7_ molecular formula. NMR measurements ^1^H NMR; ^13^C APT; edHSQC; selTOCSY on H-2 and H-3; edHSQC CH_2_ section with insert on H-2; edHSQC CH_2_ section with inserted on H-2 and H-3; and HMBC + Me section (Supporting Information, Figures S26–S32) resulted in chemical shifts very similar to those measured for compound **133**, except for the O–C(2)-H and O–C(3)-H methynes in the immediate vicinity of the −OAc group. Strong deshielding for this compound appearing on the HC-2 signals (δH-2 4.84q ^3^*J* ∼ 3.5 Hz and δC-2 73.2 ppm) evidenced the position C-2 of the OAc group. Line broadening was observed on the signals δH_3_-19 = 1.13; δC-19 = 31.4, δC-4 = 35.8, and δC-1 = 34.0 ppm. Their assignments were also supported by the selTOCSY and edHSQC experiments (Supporting Information, Figures S29–S31).

The isolation and structure elucidation of compound **135** (cyanosterone A) has already been reported with a partial NMR signal assignment in C_5_D_5_N (300/75 MHz).^35^ In the current study, we complete the available data. The HR-MS spectrum (Supporting Information, Figure S33) supported the molecular formula of C_29_H_44_O_7_. To achieve complete ^1^H and ^13^C NMR assignments (see Table 1), the following spectra were used: ^1^H NMR; ^13^C DEPTQ; HSQC; edHSQC CH_2_ section; HMBC+Me section; selROE on H_3_-18 and H_3_-19; and selTOCSY and selROE on H-5 (Supporting Information, Figures S34–40). It is worth mentioning that the selROE and selTOCSY measurements made possible a selective, nonoverlapping observation of the ^1^H signals that are sterically close to the selected H_3_-18, H_3_-19, and H_α_-5 hydrogens and those that are in spin–spin coupling with H-5.

**Table 2:** Intersection: the Number of Common Compounds Identified^a^

			similarity (%)	
1st	2nd	intersection	1st to 2nd	2nd to 1st
CAPR1	CAPR2	52	49	79
CAPR1	CARO	41	39	80
CAPR1	CALF	32	30	71
CAPR1	20EOX	33	31	67
CAPR2	CARO	30	45	58
CAPR2	CALF	21	32	46
CAPR2	20EOX	15	23	31
CARO	CALF	37	73	82
CARO	20EOX	2	4	4
CALF	20EOX	1	2	2

a Similarity 1st to 2nd, and 2nd to 1st are expressed as the percentage of detectable peaks in the 1st sample that are also present in the 2nd, and vice versa, respectively.

**Figure 2:**
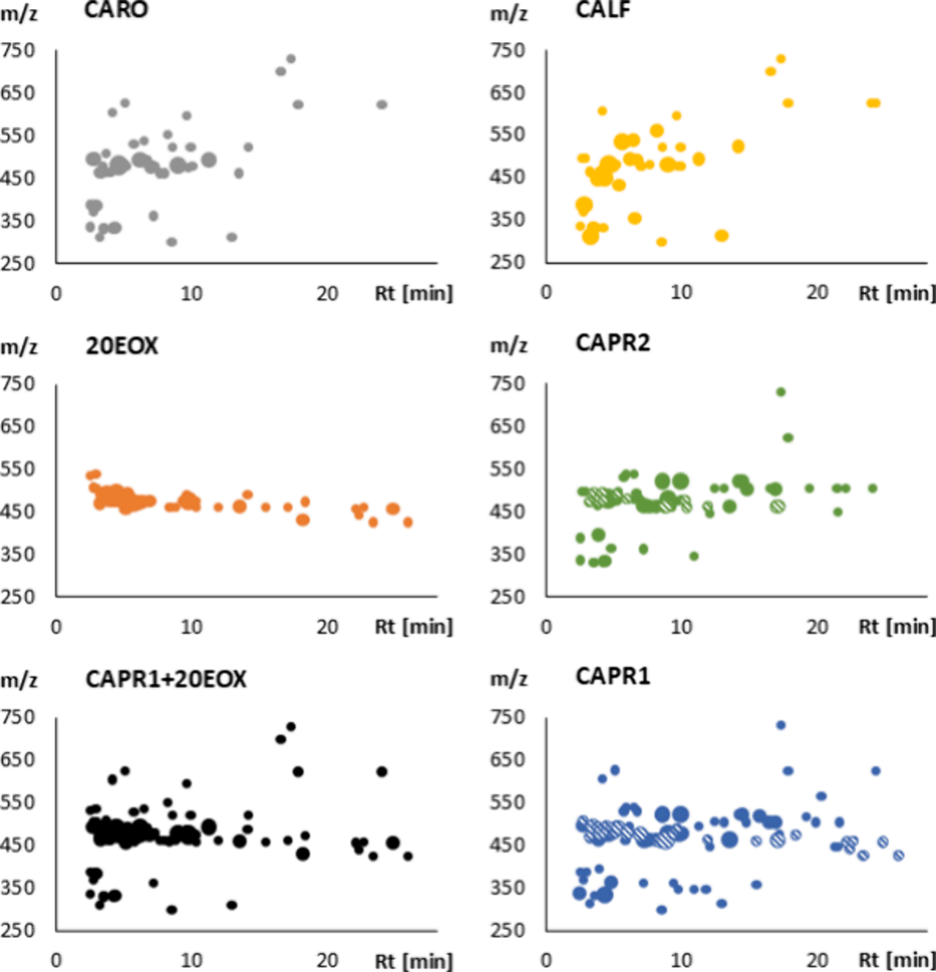
Overview of the LC-MS fingerprints of the samples analyzed. CARO: authentic *Cyanotis arachnoidea* root extract, CALF: authentic *C. arachnoidea* leaf extract, 20EOX: autoxidized product mixture of 20E, CAPR1, and CAPR2: commercially obtained *C. arachnoidea* root extracts, and CARO+20EOX: mathematical sum of CARO and 20EOX. The size of the bullets represents the relative peak area values of each peak. Striped bullets represents the presumable artifacts.

In the authentic root and leaf extracts of *C. arachnoidea* (CARO and CALF), approximately 50–60 components were detectable using the specified HPLC method. Compounds to which we did not have authentic and fully characterized reference material were tentatively identified as ecdysteroids based on their chromatographic behavior, *m*/*z* values, fragmentation patterns, and isotope distribution. The main component was 20E in both CARO and CALF. Their qualitative composition is very similar (Table 2, Figure 2), and the root extract is unsurprisingly richer in ecdysteroids than the leaf extract. It may be worth mentioning that commercially available *Cyanotis* extracts are usually declared to be root extracts.

The first step in the evaluation of the CAPR1 extract was to compare it to the authentic root extract (CARO). Obvious differences in the number of components and a shift of the major constituents toward relatively less polar derivatives were observed in the CAPR1 extract. In contrast to the 50–60 detectable components of the authentic extract, 106 components could be detected in the CAPR1 extract. For the substances we analyzed, more than 80% of the compounds found in the authentic root extract (CARO) were also detected in the CAPR1 extract, indicating that these compounds are likely to have originated genuinely from the plant. However, less than 40% of all CAPR1-detected components was found in the CARO extract. On one hand, it is important to state that the geographical origin and harvesting time of the commercial and authentic plant samples were different, which should also manifest itself in qualitative and quantitative differences in their ecdysteroid compositions. On the other hand, it seems highly unlikely that so many genuine compounds would be present only in industrially processed plants while completely missing in those collected in the wild. Instead, artifact formation should be a much more reasonable explanation for such a difference in composition.

When comparing the components of the CAPR1 extract with those of 20EOX obtained from 20E autoxidation, we found that almost 70% of the auto-oxidation products were detectable in CAPR1. This suggests the formation of oxidative artifacts during industrial processing. It is also worth stressing that (i) not only 20E, but any ecdysteroid with a 7-ene-6-one moiety in its B-ring is prone to autoxidation, and (ii) this autoxidation requires a strong alkaline medium and does not occur at neutral pH. Since all components of the 20EOX sample originated exclusively from 20E and from no other compounds present in the authentic extract, oxidation of the latter is expected to result in a more complex component profile. This may explain the large number of components detected in the CAPR1 extract, in contrast with the ca. 50 detectable oxidized derivatives in 20EOX. Additionally, since we did not use any added base at any extraction step, no such artifacts were detectable in the authentic extracts of CARO or CALF.

In contrast to CAPR1, the composition of the CAPR2 extract was closer to that of authentic CARO (Figure 2). However, CAPR2 also contained a significant proportion of compounds that are indicative of possible artifact formation. This sample was particularly rich in acetates (see Supporting Information Table S1), which may indicate the use of acetic acid at some point in the processing.

### 3.1 LC-MS Data of Identified Ecdysteroids

**Figure 3:**
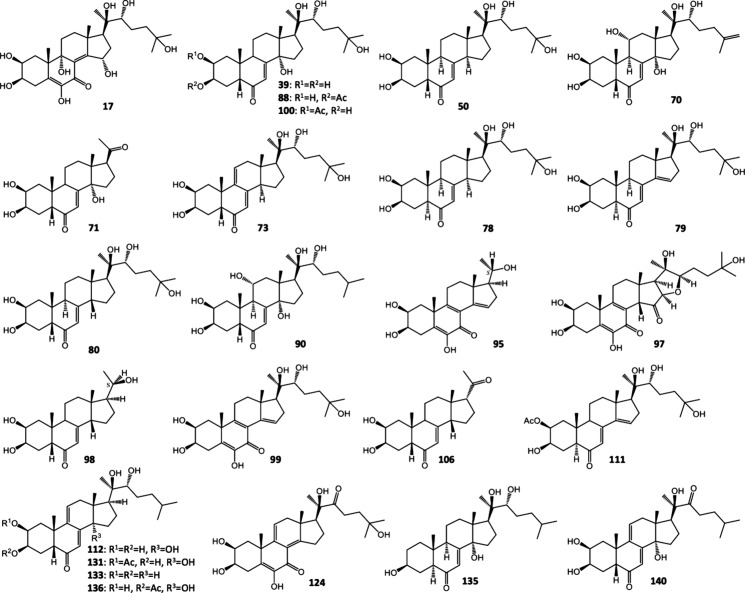
Structures of ecdysteroids unambiguously identified in the extracts using fully characterized reference standards. Compounds **79**, **111**, **131**, and **136** are new ecdysteroids reported here for the first time.

Among the vast number of ecdysteroids observed in the analyzed samples, several of them could be identified with authentic reference materials that were isolated and fully characterized in our previous studies or during the current work. The structures of these compounds and their LC-MS data are presented in Figure 3 and Table 3, respectively.

**Table 3:**
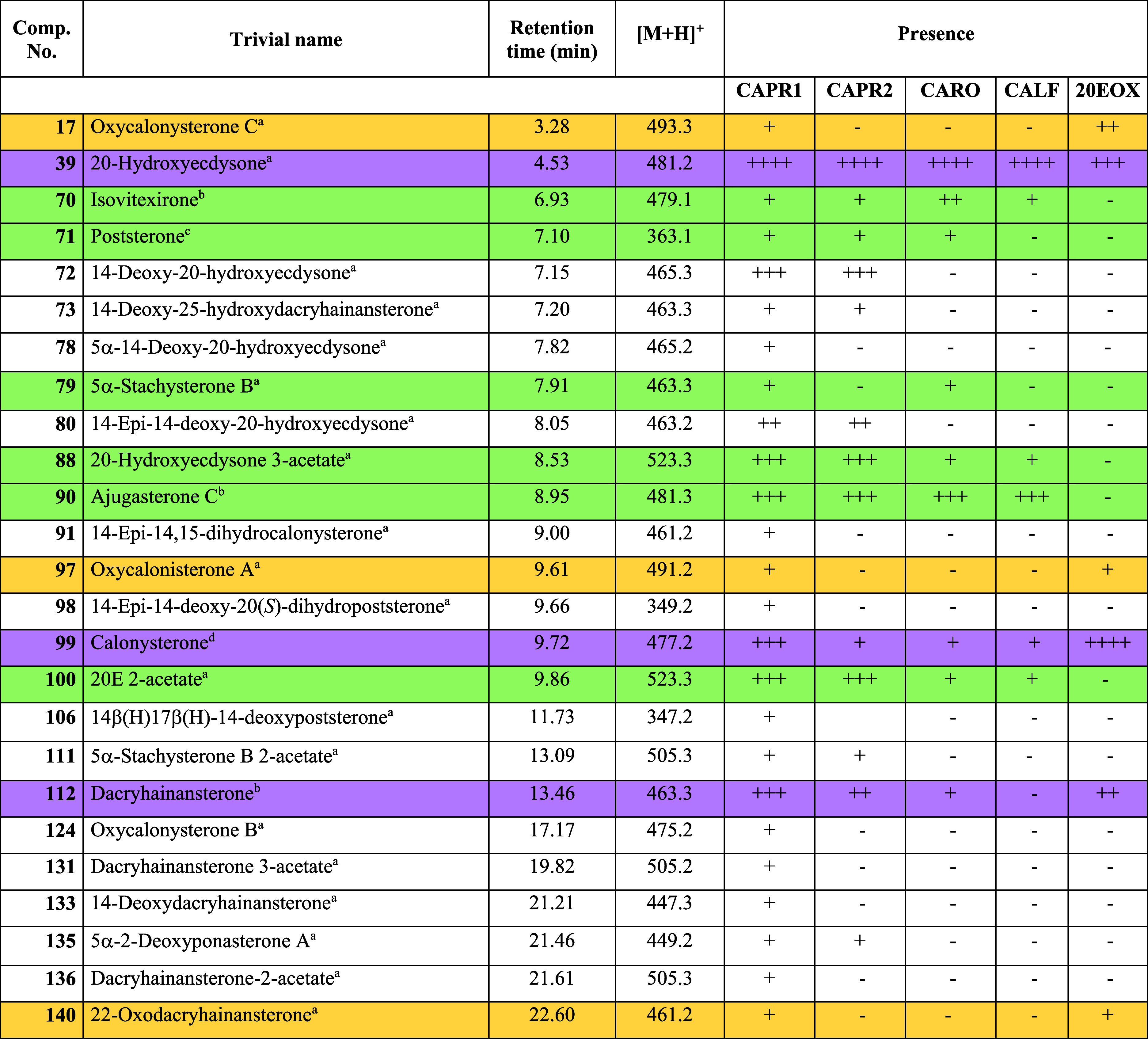
HPLC-MS Characteristics of 20 Ecdysteroids Identified by Fully Characterized Reference Standards and Their Semiquantitative Presence in the Samples^e^

a Isolated in our previous phytochemical work on commercial *Cyanotis arachnoidea* extracts.^12,30,36,37^

b Isolated in our previous phytochemical work on *Serratula wolffii* Andrae.^29^

c Prepared semisynthetically from 20E.^38^

d Obtained from the autoxidation of 20E.^28^

e Color codes for the suggested origin: green, genuine compound; yellow, oxidized artifact; violet, genuine compound present in quantities likely altered by process-related oxidation. The genuine or artifact nature of compounds without a highlighting color cannot be decided based on the available data. For a complete overview of all detected compounds, see Supplementary Information and Table S2.

There is a striking difference between authentic and industrial *C. arachnoidea* extracts in terms of their qualitative and quantitative ecdysteroid profile, and much of this gap can be filled with compounds present in the autoxidized sample (see Figure 2). It is well known that, in addition to genetic and epigenetic factors, external factors such as soil, climate, weather, and water availability can largely influence the secondary metabolite content of a plant. However, we cannot ignore the many findings in our work that all point toward process-related artifact formation. Genetic and environmental factors typically result in quantitative variations. In this work, we found the systematic appearance of a whole series of known oxidized artifacts (e.g., oxycalonysterones A and C) and tentatively identified ecdysteroids in the autoxidized mixture and the industrial samples, while undetectable in the authentic collected plant samples. It is also interesting to see the extreme deviations from expectable quantitative patterns, mainly the 20E vs calonysterone ratio. Calonysterone (**99**) is a rare natural ecdysteroid; to the best of our knowledge, it has never been isolated from any plants in larger amounts. Unsurprisingly, in both authentic root and leaf extracts, this ratio is around 500–1000:1. However, the 20E:calonysterone ratio is 10:1 in CAPR1. Altogether, our results suggest that an industrial process-related oxidative artifact formation influenced the composition of the tested commercial samples.

Identifying and isolating artifacts are crucial for comprehensive research on ecdysteroids and their derivatives. Without extensive biological studies, it is difficult to predict potential effects and side effects. Although the health benefits attributed to 20E are relatively well documented and some convincingly confirmed even in clinical studies (e.g., against COVID-19,^19^ or sarcopenia: NCT03452488), very little is known about the pharmacology of minor phytoecdysteroids in mammals. The few related studies that have been performed clearly show that the high chemical diversity of ecdysteroids does manifest in a similarly diverse pharmacology. In our previous work, we have shown that less polar ecdysteroids, such as, e.g., acetonides, exert opposite effects compared to 20E on the drug resistance in cancer cells.^39^ Autoxidized derivatives of 20E, including the major product **99**, showed a much stronger effect in activating protein kinase B (Akt) than their parent compound. However, the desmotropic counterpart of **99**, isocalonysterone (not identified in this study), exerted an opposite effect and acted as an Akt inhibitor at the tested concentration.^28^ Some other oxidized analogs of 20E were also more potent on both Akt and AMPK,^40^ which are crucial mechanisms in regulating cell death and survival.^41,42^ We recently reported oxycalonisterones A (**97**), B (**124**), and C (**17**) as potent blood–brain barrier protective agents in vitro against oxidative stress and inflammation,^37^ but as of now, no related information is available about such bioactivity of (other) natural ecdysteroids. Nonetheless, we did identify some semisynthetic ecdysteroid oxime derivatives that may dose-dependently sensitize the BBB to oxidative stress that may confer them harmful effects in this regard. This suggests that it is not straightforward to assume that all ecdysteroids will have a central nervous system protective effect and urges further study. The bioactivity profile of calonysterone (**99**) in rats also showed several differences to that of 20E. Both compounds prevented HFHSD-induced obesity and most symptoms of metabolic syndrome but were different in their potency on superoxide dismutase and catalase levels, as well as on interleukin-6 expression both on the mRNA and protein levels. These examples demonstrate that significant alteration in the ecdysteroid composition of a plant extract has a high potential to significantly change the overall bioactivity profile. This may pose both opportunities and threats. On the one hand, an appropriate and well-designed industrial processing may improve the pharmacological potential of ecdysteroid-containing herbal extracts as well as their drug discovery value through the many new compounds that may be developed as leads themselves. On the other hand, practically nothing is known about the toxicological implications of the large number of process-related artifacts, and, considering the significant market for such food supplements, this is concerning. Such implications for our results urge further related studies on the efficacy and safety of minor ecdysteroids unknowingly consumed by people worldwide including, but not limited to, athletes and body builders.

## Supporting Information Available

(Methods S1) Isolation of compounds **79**, **111**, **131**, **135**, and **136**; (Table S1) HPLC-MS characteristics and semiquantitative presence of all detected ecdysteroids in the samples; (Table S2) LC-MS/MS data of ecdysteroid compounds with authentic standards; and (Figures S1–S41) HR-MS and NMR data of new compounds (PDF)
