# Supplementary material for: LC-MS/MS Fingerprinting Analysis of Cyanotis arachnoidea Extracts: Process-Related Artifacts in Anabolic Food Supplements
Source: ACS Omega. 2025 May 2;10(18):18605–14. doi: 10.1021/acsomega.4c10908 (PMC12079227; doi:10.1021/acsomega.4c10908)

Supporting Information

Article title: LC-MS/MS fingerprinting analysis of Cyanotis arachnoidea extracts: process-related artefacts in anabolic food supplements
Authors: Dávid Laczkó, En-Liang Chu, Ching-Chia Chang, Fang-Rong Chang, Gábor Girst, Tamás Gáti, Gábor Tóth, Árpád Könczöl, Attila Hunyadi

The following Supporting Information is available for this article:

Methods S1 Isolation of compounds **79**, **111**, **131**, **135**, and **136**

An aliquot of 5460 g of CAPR1 was extracted with 15.5 L of MeOH in a percolator and evaporated to dryness under reduced pressure to yield 1398 g of dry residue. This was divided into two equal parts, each of which was fractionated by column chromatography as follows, and the corresponding fractions eluted with the same solvent were subsequently joined. Each of the two ca. 700 g of extracts was adsorbed on 2100g of silica and applied on top of a column of 1500 g of silica. Elution was performed by CH2Cl2 (20 L) followed by a stepwise gradient of CH2Cl2 – MeOH mixtures (97:3, 10 L; 95:5, 20 L; 93:7, 22.5 L; 85:15, 25 L). The combined fractions eluted with 95:5 (F1), 93:7 (F2), and 85:15 (F3) were evaporated to dryness to yield 210, 415.8, and 406 g of dry residues, respectively.

Fraction F1 was adsorbed to 840 g of Celite 545 and layered on top of a 2540 g silica column. Gradient elution was performed, and a total of 80 fractions (F1.1-80) of 2.5 L each were collected, using *c*-hexane (F1.1), *c*-hexane – ethyl acetate (6:2, F1.2-3), (6:3, F1.4-5), (6:4, F1.6-9), (6:5, F1.10-19), and *c*-hexane – ethyl acetate – ethanol (60:50:2, F1.20-30), (60:50:5, F1.31-49), (60:50:10, F1.50-66), (60:50:20, F1.67-80) solvent systems. Fractions F1.26-51 were combined evaporated to dryness to yield 130.8 g of dry residue. This was adsorbed on 327 g of RediSep Rf C18 (40-60 µm, Teledyne Isco Inc, Lincoln, NE, USA) and applied on top of 400 g of the same stationary phase. Gradient elution was performed, and a total of 35 fractions (F1.RP1-31) were collected, using H_2O – acetonitrile (8:2, F1.RP1-23), (7:3, F1.RP24-31), and (6:4, F1.RP32-35) solvent systems. The dry residue of fraction F1.RP6–10 (29.8 g) was purified by centrifugal partition chromatography (CPC) using *n*-hexane – ethyl acetate – methanol water (3:7:3:7) biphasic solvent system in the ascending mode. The major peak was collected and crystallized to obtain compound **111.**

The combined dry residue of fractions F1.RP26-31 (19.6 g) was divided into two, and each was further fractionated by flash chromatography on an 80g RediSep Silica Gold column with dry loading technique (flow rate 60 mL/min, fraction collection based on detector signal, run time: 70 min) with a gradient of CH2Cl2 (A) and *i*-PrOH (B), from 0 to 30 % of solvent B in A. The corresponding fractions of these two separations were joined into 5 sub-fractions (F1.RP26-31.NP1-5).

F1.RP26-31.NP2 (3.06 g) was further purified by flash chromatography on a 24 g RediSep Silica Gold column (flow rate 35 mL/min, fraction collection based on detector signal, run time: 75 min) with a gradient of n-hexane - CH_2Cl_2 (1:1; A) and 96% ethanol (B), from 3 to 10 % of solvent B in A. Fractions 26–30 were joined and purified by CPC using *n*-hexane – ethyl acetate – methanol water (1:1:1:1) biphasic solvent system in the ascending mode. The first major peak was collected and further purified by semipreparative NP-HPLC on a Zorbax Sil (9.4x250 mm, 5 µm) column using CH_2Cl_2 – isopropanol – water (125:15:1) at a flow rate of 3 mL/min to obtain compound **135**.

F1.RP26-31.NP3 (1.70 g) was further purified by flash chromatography on a 24 g RediSep Silica Gold column (flow rate 35 mL/min, fraction collection based on detector signal, run time: 75 min) with a gradient of *n*-hexane - CH_2Cl_2 (1:1; A) and 96% ethanol (B), from 2 to 5 % of solvent B in A. The major peak was purified by preparative RP-HPLC on a Kinetex XB-C18 (21x250 mm, 5 µm) column, using isocratic elution with 37% aqueous acetonitrile at a flow rate of 15 mL/min to obtain compound **131**. A partially resolved impurity peak was also collected, and semipreparative NP-HPLC was used on a Zorbax Sil (9.4x250 mm, 5 µm) column using CH_2Cl_2 – isopropanol – water (125:15:1) at a flow rate of 2.5 mL/min to obtain compound **136**.

Fraction F2 (415.8 g) was adsorbed on 1000 g of silica, applied on top of a column of 2000 g of silica, and further fractionated with a stepwise gradient of *n*-hexane - ethyl acetate - ethanol, 60:40:0, 60:50:0, 60:50:2, 60:50:5, 60:50:10, 60:50:20, and 60:50:30, collecting 350 fractions of 500 mL each (F2.1-350).

Fractions F2.115-153 (78.9 g) were joined, adsorbed on 300 g of RediSep Rf C18 (40-60 µm, Teledyne Isco Inc, Lincoln, NE, USA), applied on top of 500 g of the same stationary phase, and further fractionated with aqueous methanol (10%, fr. 1-37; 15%, fr. 38-50; 20%, fr. 51-60; 25%, fr. 61-83; 30%, fr. 84-97; and 40%, fr. 98-127). Fractions 91-105 (10.4 g), 106-115 (7.7 g), and 116-127 (4.9 g) were separately purified by flash chromatography (dry loading on 20 g of silica, 330 g RediSep Silica Gold column, solvent A: CH_2Cl_2, solvent B: 99% methanol(aq), gradient: 4% B in A increasing to 10% in 60 min). Fractions were collected based on detector signal, and fraction 7 (2.5g was purified by preparative RP-HPLC (Kinetex XB-C18, 21x250 mm, 5µm; 23% ACN(aq), flow rate: 15 mL/min), and subsequently by preparative SFC (Luna Silica, 21x250 mm, 5µm; 20% EtOH in CO_2, flow rate: 15 mL/min) to afford compound **79**.

Table S1 LC-MS/MS data of ecdysteroid compounds with authentic standards. Color codes for the suggested origin of compounds in the commercial extracts (CAPR1 and CAPR2): green, genuine compound; yellow, oxidized artefact; violet, genuine compound present in quantities likely altered by oxidation. Compounds without a highlighting color are either not detectable in the commercial extracts, or their genuine or artefact nature cannot be decided based on the available data.

Trivial name	tR (min)	UV max	EM	Precursor ion	Fragment ions (int. %)	DP (V)	CE (V)	CAV (V)	Chemical structure
5α-Stachysterone B 2-acetate	13.09	300.8	504.3	505.3	343 (100,0) 451,1 (39,5) 283,2 (14,7) **505,3 (12,8)** 469,1 (11,8) 335,1 (5,1) 340,9 (4,8) 391,1 (4,5) 394,9 (3,7) 127,3 (3,7)	135	15	4	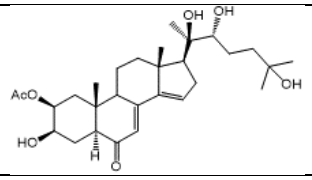
Dacryhainansterone 2-acetate	19.82	302.1	504.2	505.2	341 (100,0) 281 (76,3) 391,1 (53,4) 469,4 (41,3) **505,2 (32,3)** 409,2 (27,8) 445 (27,5) 342,9 (18,1) 293,3 (14,8) 450,7 (14,2)	135	15	4	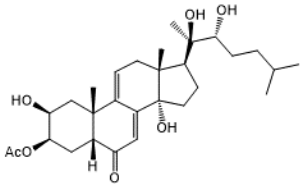
Dacryhainansterone 3-acetate	21.61	302.1	504.3	505.3	341,1 (100,0) **505,3 (87,4)** 451,2 (66,7) 281 (30,2) 469 (19,5) 342,9 (14,0) 283 (13,3) 409,1 (11,1) 308,9 (10,3) 391,3 (10,1)	135	15	4	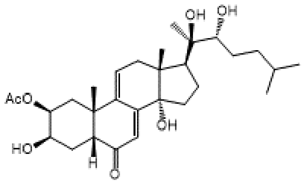
14-Deoxydacryhainanstero ne	21.21	300.0	446.3	447.3	**447,3 (100,0)** 109,2 (29,3) 301,2 (27,2) 282,9 (20,6) 329,2 (16,3) 225,1 (13,7) 429,2 (12,3) 285 (11,8) 173,2 (10,3) 227,2 (10,0))	135	25	4	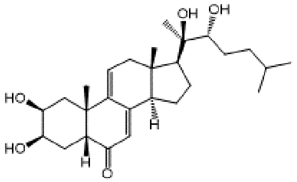
2-Deoxy-5α-Ponasterone A	21.46	243.9	448.2	449.2	413,3 (100,0) 431,3 (73,5) 430,9 (51,4) **449,2(30,6)** 302,9 (28,6) 181 (20,2) 297 (18,7) 314,8 (16,9) 188,8 (15,7) 151,4 (15,6)	135	15	4	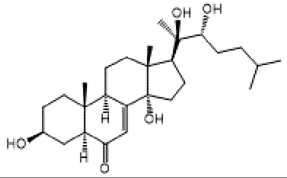

22-Oxo-dacryhainansterone	22.6	302.3	460.2	461.2	299,1 (100,0) **461,2 (34,7)** 425 (26,1) 179,1 (24,7) 443,1 (15,0) 231,1 (11,5) 301 (8,6) 281,1 (7,7) 407,2 (7,5) 248,9 (7,3)	135	20	4	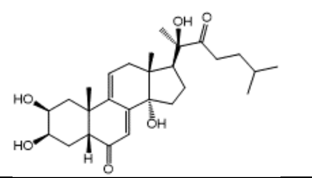
Isovitexirone	6.93	246.0	478.1	479.1	443 (100,0) 425,1 (74,1) **479,1 (63,0)** 299 (53,4) 327,1 (48,9) 407,1 (33,7) 460,9 (31,2) 387,1 (30,3) 165 (19,8) 281,1 (16,9)	135	15	4	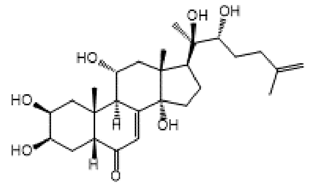
14-deoxy-25-hydroxydacryhainansterone	7.2	302.9	462.3	463.3	299,1 (100,0) 427,3 (69,2) **463,3 (64,1)** 283,1 (39,1) 445,2 (36,5) 301,1 (34,0) 125 (24,5) 225,1 (17,5) 409,2 (17,2) 353,1 (15,1)	135	15	4	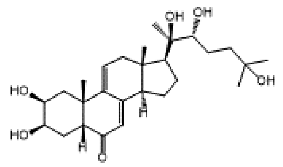
5α-Stachysterone B	7.91	301.0	462.3	463.3	301,1 (100,0) 409,1 (44,4) **463,3 (22,2)** 427 (16,4) 299,1 (8,6) 353 (7,5) 283,1 (5,8) 329,1 (5,3) 127 (4,3) 391,1 (2,5)	135	15	4	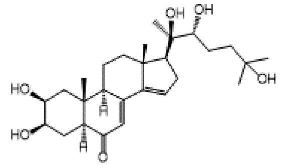
5α-14-Deoxy-20-hydroxyecdysone	7.82	250.2	464.2	465.2	285,1 (100,0) 125,1 (91,1) 109,1 (70,0) **465,2 (67,1)** 447,1 (56,2) 389,2 (50,8) 267,2 (50,1) 429,3 (27,6) 393,1 (25,7) 411,1 (24,5)	135	25	4	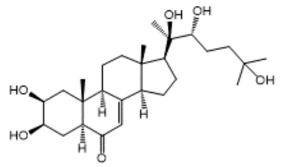
14-Epi-14-deoxy-20-hydroxyecdysone	8.05	256.1	464.3	465.3	429,1 (100,0) 447,2 (24,3) 331,1 (12,1) 411,1 (8,5) **465,3 (3,3)** 285,2 (1,9) 302,9 (1,3) 393 (1,1)	135	10	4	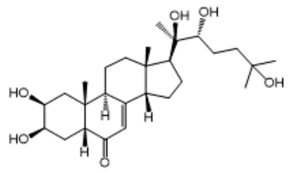
Oxycalonysterone A	9.61	200.0	490.2	491.2	473,1 (100,0) 455,1 (21,7) 259 (17,0) **491,2 (10,6)** 143,1 (9,0) 313,0 (7,5) 331 (5,8) 409,1 (4,0) 125,1 (3,7) 437,1 (3,2)	135	10	4	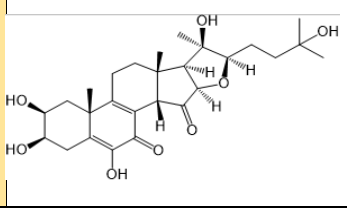

Oxycalonysterone B	17.17	222.3	474.2	475.2	113,1 (100,0) 457,1 (54,3) 439,1 (40,5) **475,2 (16,0)** 223,1 (11,1) 317,1 (9,6) 177,1 (8,8) 421 (8,2) 299,0 (5,0) 235,1 (3,9)	135	15	4	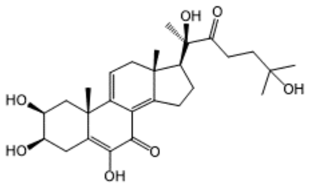
14-Epi-14-deoxy-20(*S*)-dihydroposterone	9.66	256.1	348.2	349.2	331,1 (100,0) **349,2 (89,4)** 109 (86,7) 135,1 (84,3) 121,1 (80,6) 191,1 (77,5) 173 (73,8) 313,1 (65,2) 107,1 (55,8) 209,1 (51,1)	135	15	4	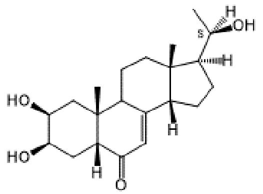
14-Dihydro-14β(H)17β(H)-poststerone	11.73	252.1	346.2	347.2	329,1 (100,0) 271,1 (94,9) 311,1 (91,2) **347,2 (81,1)** 293,1 (37,1) 253,1 (20,0) 237 (14,0) 173 (13,5) 241,2 (11,9) 255 (11,7)	135	15	4	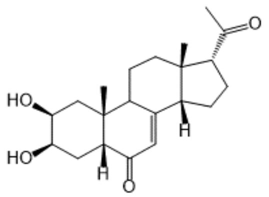
20-Hydroxyecdysone 2-acetate	9.86	245.4	522.3	523.3	487,2 (100,0) 413,1 (98,9) 469,1 (52,5) **523,3 (47,0)** 427,1 (36,3) 345 (31,5) 505,3 (15,6) 409,2 (14,0) 445,1 (12,2) 463 (3,9)	135	10	4	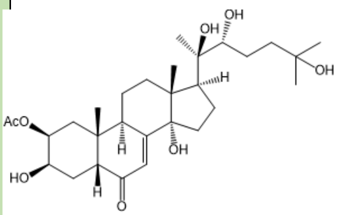
20-Hydroxyecdysone 3-acetate	8.53	245.4	522.3	523.3	487,2 (100,0) 413,2 (84,9) 469,1 (50,2) 427,1 (32,1) **523,3 (31,3)** 345 (29,9) 505 (29,8) 409,2 (15,3) 445 (5,3) 165,1 (5,2)	135	10	4	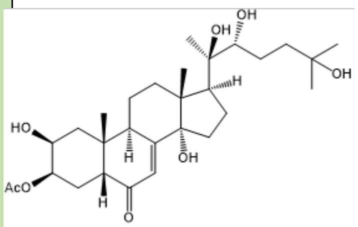
Ajugasterone C	8.95	245.0	480.3	481.3	427,1 (100,0) 445,2 (86,3) **481,3 (60,4)** 409 (59,1) 299,1 (48,7) 310,9 (40,6) 463,1 (40,1) 281,2 (26,7) 329 (17,0) 391,2 (16,9)	135	15	4	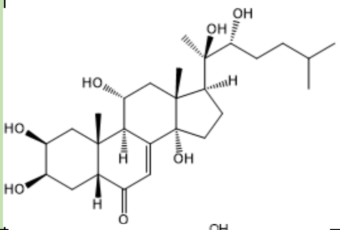
Calonysterone	9.72	224.1	476.2	477.2	459,1 (100,0) 441,1 (79,8) **477,2 (34,3)** 423,1 (31,1) 223 (24,8) 317 (23,4) 343 (17,0) 177,2 (16,4) 299,1 (14,6) 367 (9,8)	135	15	4	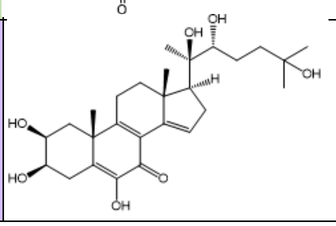

Dacryhainansterone	13.46	304.1	462.3	463.3	299,2 (100,0)<br>409,2 (72,5)<br>109,1 (29,6)<br>301,2 (14,9)<br>281,2 (10,2)<br>231,2 (10,1)<br>145,1 (9,9) 327,2<br>(9,9) **463,3 (9,9)**<br>427,2 (9,9)	135	25	4	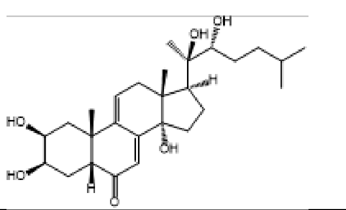

Table S2 HPLC-MS characteristics and semiquantitative presence of all detected ecdysteroids in the samples. Color codes for the suggested origin of compounds in the commercial extracts (CAPR1 and CAPR2): green, genuine compound; yellow, oxidized artefact; violet, genuine compound present in quantities likely altered by oxidation. Compounds without a highlighting color are either not detectable in the commercial extracts, or their genuine or artefact nature cannot be decided based on the available data.

Comp. No.	Trivial name, if available	Authentic standard	Retention time (min)	[M+H]$^+$	MW	Presence				
						CAPR1	CAPR2	CARO	CALF	20EOX
1	n.a.	-	2.45	337.2	336.2	++	+	+	+	-
2	n.a.	-	2.47	535.3	534.3	-	-	-	-	+
3	n.a.	-	2.50	389.2	388.2	+	+	+	+	-
4	n.a.	-	2.60	497.2	496.2	++	+	++	+	-
5	n.a.	-	2.67	509.2	508.2	+	-	-	-	+
6	n.a.	-	2.68	497.2	496.2	-	+	+	-	-
7	n.a.	-	2.72	387.0	386.0	-	-	+	+++	-
8	n.a.	-	2.73	371.1	370.1	+	-	+	+	-
9	n.a.	-	2.80	497.4	496.4	+	+	+	+	-
10	n.a.	-	2.82	497.4	496.4	+	+	++	+	-
11	n.a.	-	2.87	539.4	538.4	-	-	-	-	+
12	n.a.	-	2.91	387.1	386.1	+	-	++	++	-
13	n.a.	-	2.97	507.2	506.2	-	-	-	-	+
14	n.a.	-	3.15	473.2	472.2	+	+	-	-	++
15	n.a.	-	3.20	313.0	312.0	+	-	+	+++	-
16	n.a.	-	3.21	465.2	464.2	+	-	++	+	-
17	n.a.	+	3.28	493.3	492.3	+	-	-	-	++
18	n.a.	-	3.37	495.3	494.3	++	-	-	-	+
19	n.a.	-	3.39	333.2	332.2	-	+	+	++	-
20	n.a.	-	3.41	481.2	480.2	+	+	+	-	-
21	n.a.	-	3.46	495.2	494.2	-	+	-	-	+
22	n.a.	-	3.50	333.1	332.1	+	+	+	++	-
23	n.a.	-	3.60	511.1	510.1	-	-	+	-	-
24	n.a.	-	3.66	495.3	494.3	+	-	-	-	+++
25	n.a.	-	3.74	495.2	494.2	+	-	-	-	+
26	n.a.	-	3.75	449.1	448.1	-	-	-	+++	-
27	n.a.	-	3.76	465.1	464.1	+	+	+	-	-
28	n.a.	-	3.84	397.0	396.0	+	++	-	-	-
29	n.a.	-	3.85	465.2	464.2	++	-	+	+	-
30	n.a.	-	3.92	481.2	480.2	++	++	-	-	+++
31	n.a.	-	3.94	465.1	464.1	+	-	+	+	-
32	n.a.	-	4.08	607.3	606.3	+	-	+	+	-
33	n.a.	-	4.09	495.1	494.1	-	+	-	-	++
34	n.a.	-	4.15	333.0	332.0	+	+	+	+	-
35	n.a.	-	4.20	479.2	478.2	+	+	-	-	++
36	n.a.	-	4.28	449.1	448.1	-	-	-	+++	-
37	n.a.	-	4.31	335.2	334.2	+++	++	++	-	-
38	n.a.	-	4.37	497.3	496.3	+	-	-	-	+++
39	20-hydroxyecdysone	+	4.53	481.2	480.2	++++	++++	++++	++++	+++

**40**	n.a.	-	4.70	495.3	494.3	+	-	-	-	+
**41**	n.a.	-	4.73	365.1	364.1	++	+	-	-	-
**42**	n.a.	-	4.88	479.3	478.3	+	-	-	-	+
**43**	n.a.	-	4.89	481.2	480.2	+	++	++	++	-
**44**	n.a.	-	4.96	495.2	494.2	-	-	-	-	+
**45**	n.a.	-	5.00	627.3	626.3	+	-	+	-	-
**46**	n.a.	-	5.02	479.3	478.3	+	-	-	-	+
**47**	n.a.	-	5.10	459.2	458.2	-	-	-	-	++
**48**	n.a.	-	5.16	481.3	480.3	+	++	+	+	-
**49**	n.a.	-	5.23	493.2	492.2	++	+	-	-	++
**50**	n.a.	-	5.24	497.1	496.1	+	+	-	-	-
**51**	n.a.	-	5.31	433.2	432.2	-	-	-	++	-
**52**	n.a.	-	5.53	535.2	534.2	-	-	-	+++	-
**53**	n.a.	-	5.56	479.2	478.2	-	-	-	-	++
**54**	n.a.	-	5.67	531.3	530.3	+	+	+	-	-
**55**	n.a.	-	5.70	461.3	460.3	-	-	-	-	+
**56**	n.a.	-	5.78	463.3	462.3	+	-	-	-	-
**57**	n.a.	-	5.83	539.1	538.1	+	+	-	-	-
**58**	n.a.	-	5.91	481.2	480.2	++	+	-	-	+
**59**	n.a.	-	5.93	473.2	472.2	-	-	-	-	++
**60**	n.a.	-	6.04	473.2	472.2	-	-	-	-	++
**61**	n.a.	-	6.10	495.3	494.3	+	-	+++	++	-
**62**	n.a.	-	6.32	475.2	474.2	-	-	-	-	++
**63**	n.a.	-	6.39	479.1	478.1	-	+	-	-	-
**64**	n.a.	-	6.40	539.1	538.1	+	+	+	++	-
**65**	n.a.	-	6.49	355.1	354.1	-	-	-	++	-
**66**	n.a.	-	6.60	495.2	494.2	-	+	+	-	-
**67**	n.a.	-	6.65	493.2	492.2	-	-	+	++	-
**68**	n.a.	-	6.66	531.1	530.1	+	-	-	-	-
**69**	n.a.	-	6.86	477.3	476.3	++	-	-	-	++
**70**	Isovitexirone	+	6.93	479.1	478.1	+	+	++	+	-
**71**	Poststerone	+	7.10	363.1	362.1	+	+	+	-	-
**72**	n.a.	-	7.15	465.3	464.3	+++	+++	-	-	-
**73**	14-deoxy-25-hydroxydacryhainansterone	+	7.20	463.3	462.3	+	+	-	-	-
**74**	n.a.	-	7.21	481.2	480.2	-	-	+	-	-
**75**	n.a.	-	7.27	461.2	460.2	+	-	-	-	-
**76**	n.a.	-	7.59	463.3	462.3	++	++	+	-	-
**77**	n.a.	-	7.61	481.2	480.2	-	-	-	+	-
**78**	5α-14-Deoxy-20-hydroxyecdysone	+	7.82	465.2	464.2	+	-	-	-	-
**79**	5α-Stachysterone B	+	7.91	463.3	462.3	+	-	+	-	-
**80**	14-Epi-14-deoxy-20-hydroxyecdysone	+	8.05	465.2	464.2	++	++	-	-	-
**81**	n.a.	-	8.13	554.0	553.0	-	-	+	-	-
**82**	n.a.	-	8.14	561.1	560.1	-	-	-	++	-
**83**	n.a.	-	8.25	461.2	460.2	+	-	-	-	+
**84**	n.a.	-	8.26	523.2	522.2	+	-	-	-	-
**85**	n.a.	-	8.35	463.2	462.2	++	-	-	-	+
**86**	n.a.	-	8.42	465.2	464.2	-	++	-	-	-
**87**	n.a.	-	8.46	300.3	299.3	+	-	+	+	-
**88**	20-Hydroxyecdysone 3-acetate	+	8.53	523.3	522.3	+++	+++	+	+	-
**89**	n.a.	-	8.76	463.3	462.3	+++	++	-	-	+
**90**	Ajugasterone C	+	8.95	481.3	480.3	+++	+++	+++	+++	-
**91**	n.a.	-	9.00	461.2	460.2	+	-	-	-	-
**92**	n.a.	-	9.13	445.2	444.2	+	+	+	+	-
**93**	n.a.	-	9.20	463.1	462.1	-	+	-	-	-
**94**	n.a.	-	9.26	477.2	476.2	+	-	-	-	+
**95**	n.a.	+	9.31	361.2	360.2	+	-	-	-	-
**96**	n.a.	-	9.54	597.3	596.3	-	-	+	+	-
**97**	Oxycalonysterone A	+	9.61	491.2	490.2	+	-	-	-	+

**98**	14-Epi-14-deoxy-20(*S*)-dihydropoststerone	+	9.66	349.2	348.2	+	-	-	-	-
**99**	Calonysterone	+	9.72	477.2	476.2	+++	+	+	+	++++
**100**	20-Hydroxyecdysone 2-acetate	+	9.86	523.3	522.3	+++	+++	+	+	-
**101**	n.a.	-	9.97	479.2	478.2	++	+	+	+	-
**102**	n.a.	-	10.21	477.3	476.3	-	+	-	-	+
**103**	n.a.	-	10.29	461.2	460.2	-	+	-	-	+
**104**	n.a.	-	10.82	347.1	346.1	+	+	-	-	-
**105**	n.a.	-	11.20	495.3	494.3	+	-	+++	++	-
**106**	14-Dihydro-14β(H)17β(H)-poststerone	+	11.73	347.2	346.2	+	-	-	-	-
**107**	n.a.	-	11.87	463.2	462.2	+	+	-	-	+
**108**	n.a.	-	12.05	447.2	446.2	+	+	-	-	-
**109**	n.a.	-	12.41	507.2	506.2	+	+	-	-	-
**110**	n.a.	-	12.89	314.2	313.2	+	-	+	++	-
**111**	5α-Stachysterone B 2-acetate	+	13.09	505.3	504.3	+	+	-	-	-
**112**	Dacryhainansterone	+	13.46	463.3	462.3	+++	++	+	-	++
**113**	n.a.	-	14.06	491.2	490.2	-	-	-	-	+
**114**	n.a.	-	14.11	523.3	522.3	+	++	+	++	-
**115**	n.a.	-	14.40	523.4	522.4	++	++	-	-	-
**116**	n.a.	-	14.72	505.2	504.2	+	++	-	-	-
**117**	n.a.	-	15.42	461.2	460.2	+	-	-	-	+
**118**	n.a.	-	15.45	359.1	358.1	+	-	-	-	-
**119**	n.a.	-	15.71	519.4	518.4	++	-	-	-	-
**120**	n.a.	-	16.43	505.4	504.4	++	+	-	-	-
**121**	n.a.	-	16.51	701.3	700.3	-	-	+	+	-
**122**	n.a.	-	16.82	505.1	504.1	++	++	-	-	-
**123**	n.a.	-	17.01	463.3	462.3	++	++	-	-	+
**124**	Oxycalonysterone B	+	17.17	475.2	474.2	+	-	-	-	-
**125**	n.a.	-	17.23	731.3	730.3	+	+	+	+	-
**126**	n.a.	-	17.79	625.3	624.3	+	+	+	+	-
**127**	n.a.	-	18.14	432.4	431.4	-	-	-	-	++
**128**	n.a.	-	18.34	475.2	474.2	+	-	-	-	+
**129**	n.a.	-	19.12	519.4	518.4	+	-	-	-	-
**130**	n.a.	-	19.40	505.2	504.2	-	+	-	-	-
**131**	Dacryhainansterone 2-acetate	+	19.82	505.2	504.2	+	-	-	-	-
**132**	n.a.	-	20.23	565.3	564.3	+	-	-	-	-
**133**	14-Deoxydacryhainansterone	+	21.21	447.3	446.3	+	-	-	-	-
**134**	n.a.	-	21.41	505.2	504.2	-	+	-	-	-
**135**	2-Deoxy-5α-Ponasterone A	+	21.46	449.2	448.2	+	+	-	-	-
**136**	Dacryhainansterone 3-acetate	+	21.61	505.3	504.3	+	-	-	-	-
**137**	n.a.	-	22.07	459.1	458.1	+	-	-	-	+
**138**	n.a.	-	22.05	505.2	504.2	-	+	-	-	-
**139**	n.a.	-	22.31	443.3	442.3	+	-	-	-	+
**140**	22-Oxo-dacryhainansterone	+	22.6	461.2	460.2	+	-	-	-	+
**141**	n.a.	-	23.32	427.2	426.2	+	-	-	-	+
**142**	n.a.	-	23.95	625.2	624.2	-	-	+	+	-
**143**	n.a.	-	24.05	505.2	504.2	-	+	-	-	-
**144**	n.a.	-	24.30	625.3	624.3	+	-	-	+	-
**145**	n.a.	-	24.75	459.2	458.2	+	-	-	-	++
**146**	n.a.	-	25.92	427.2	426.2	+	-	-	-	+

Figure S1-S41 HRMS and NMR data of new compounds.

Figure S1. Compound **79** HRMS

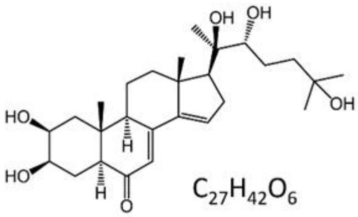

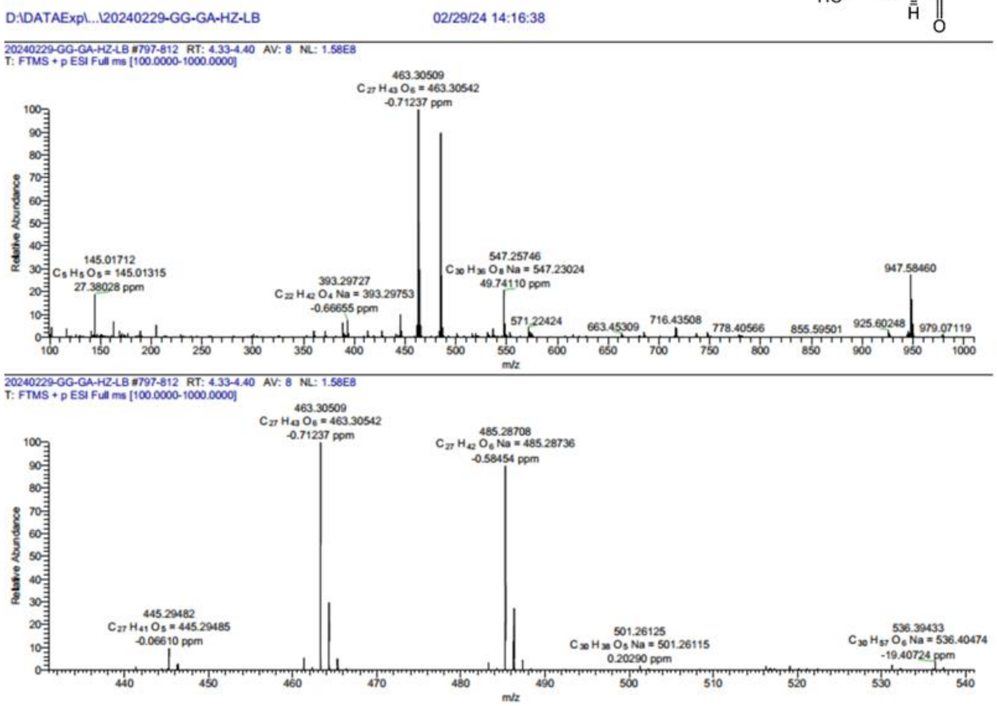

Figure S2. Compound **79** ^{1}H NMR

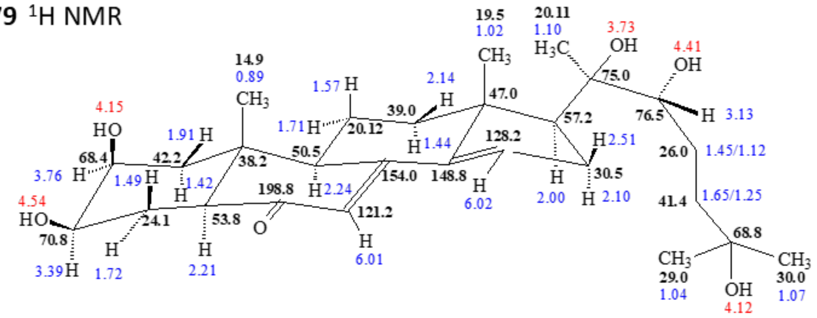

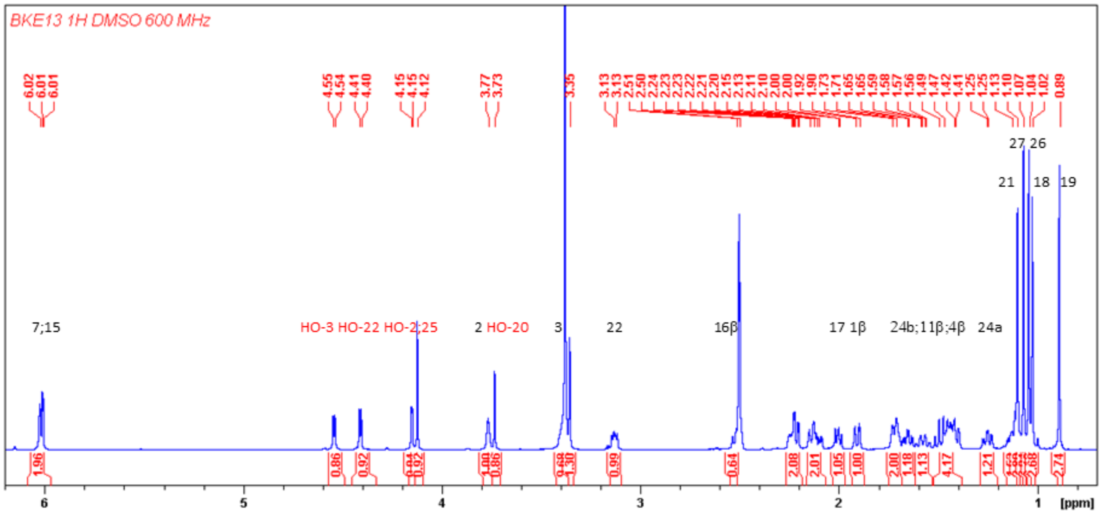

Figure S3. Compound **79**
^{13}C NMR+DEPT135

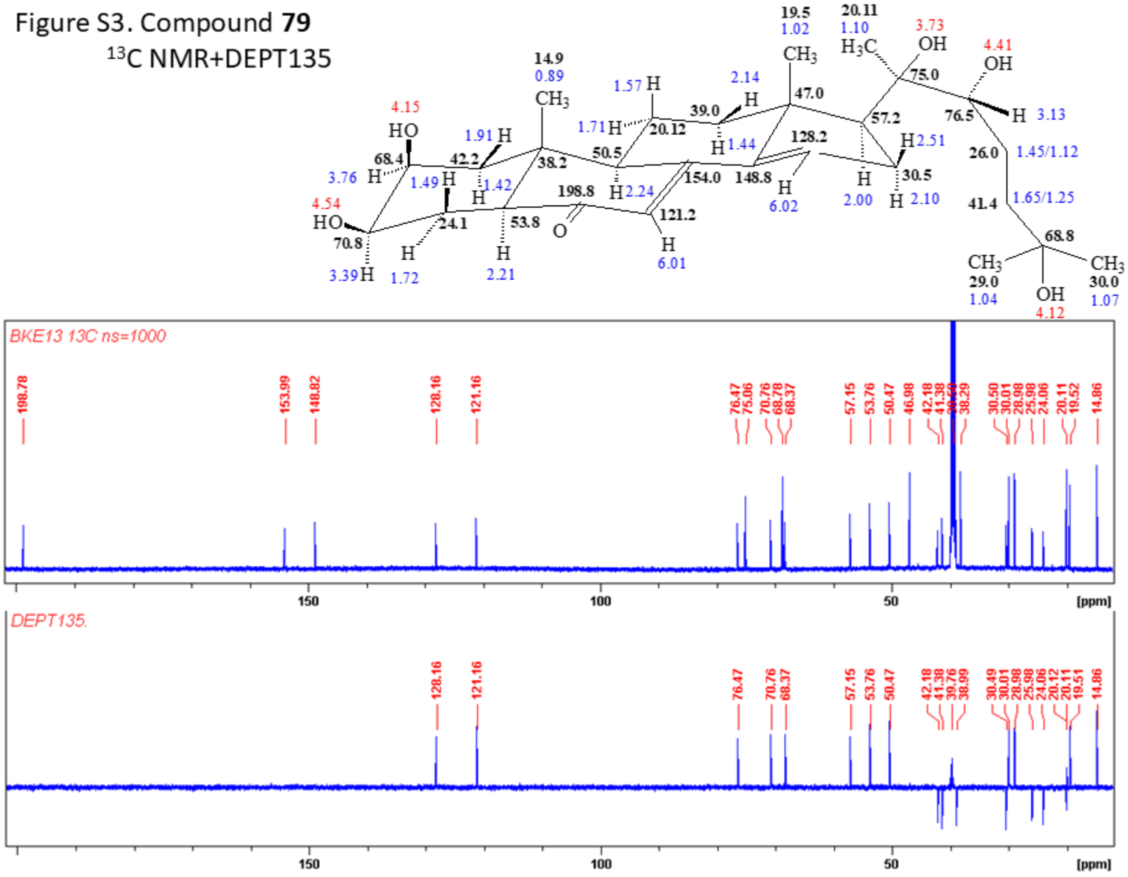

Figure S4. Compound **79** edHSQC

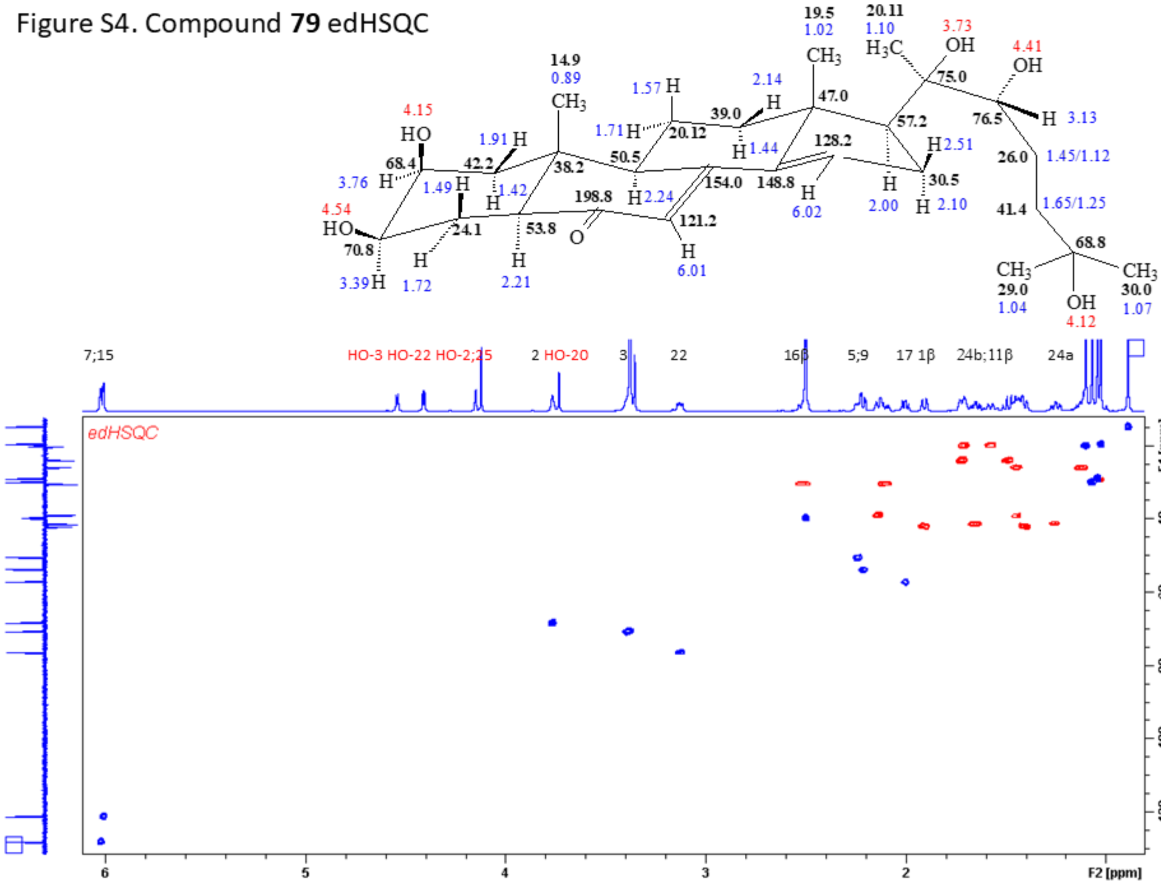

Figure S5. Compound **79** HMBC+Me-section

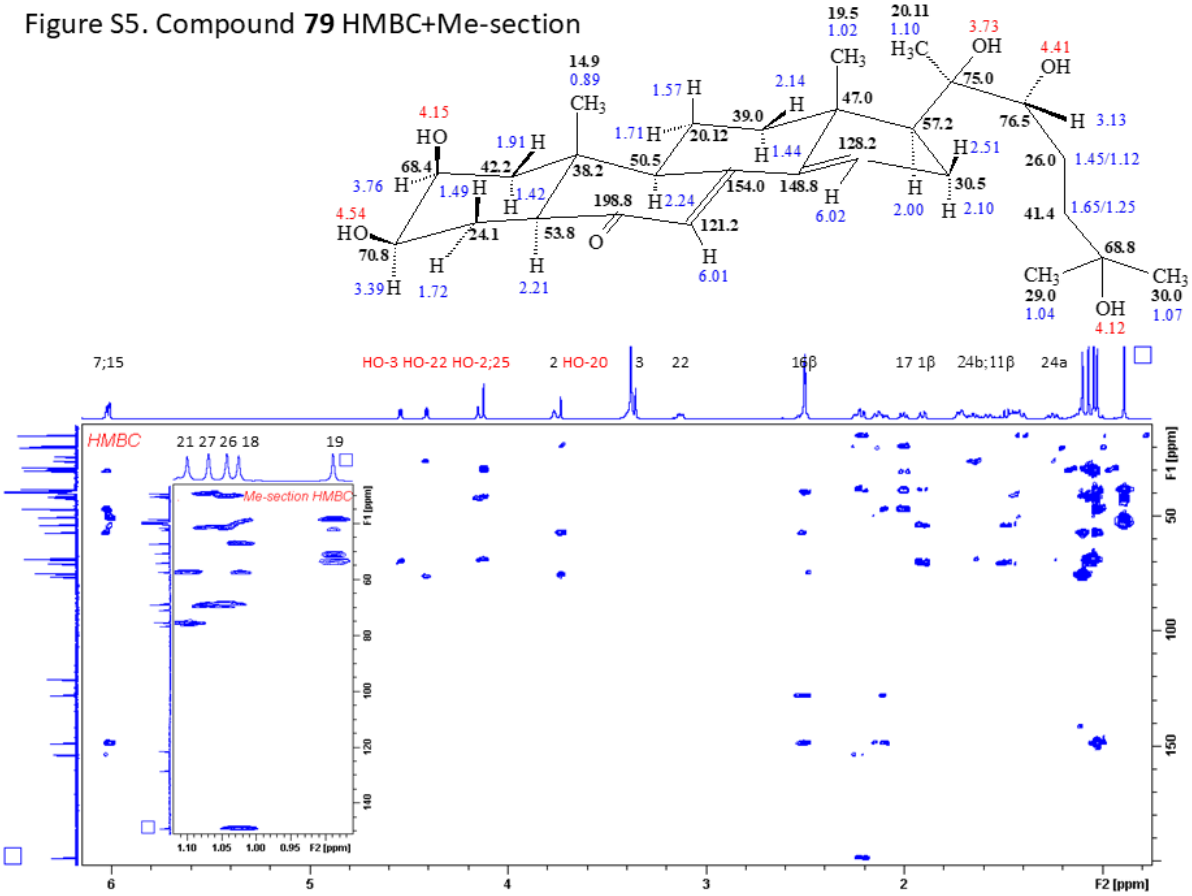

Figure S6. Compound **79** $^1H,^1H$-COSY

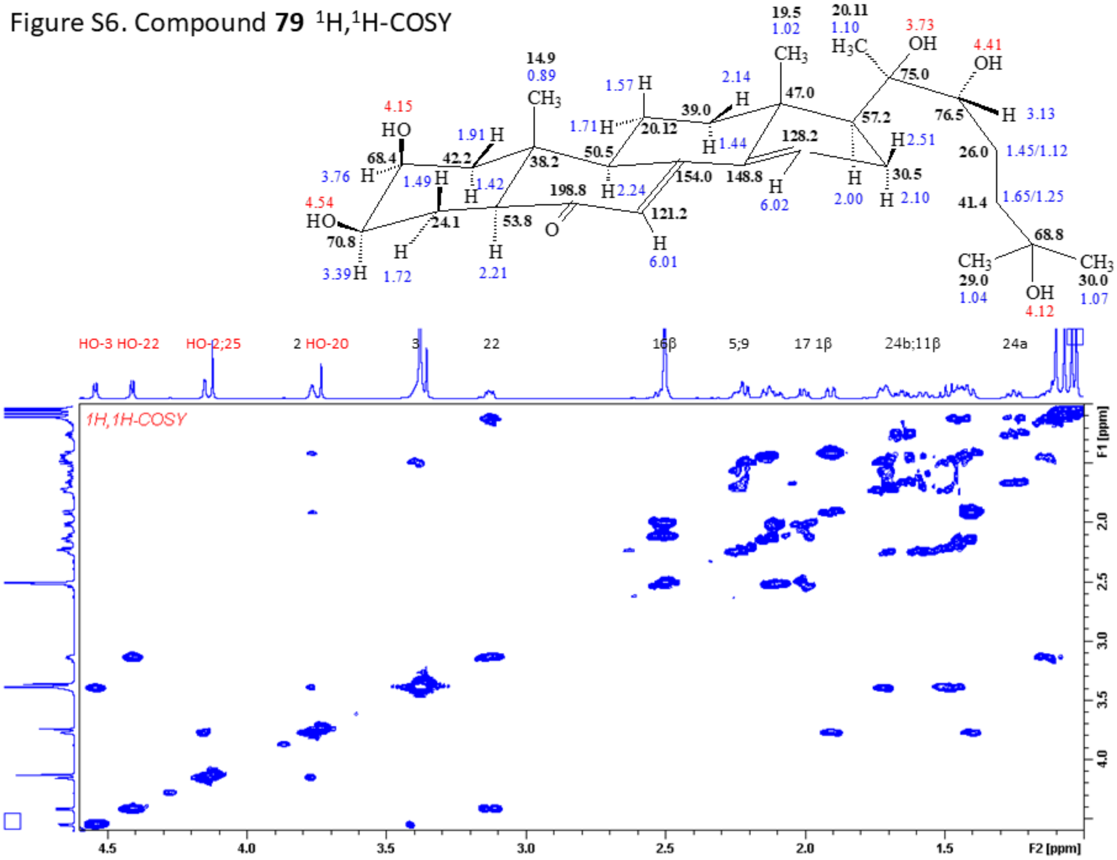

Figure S7. Compound **79** ^{1}H NMR
+ selROE on H_3-19 and H_3-18
τ_{mix}300 ms

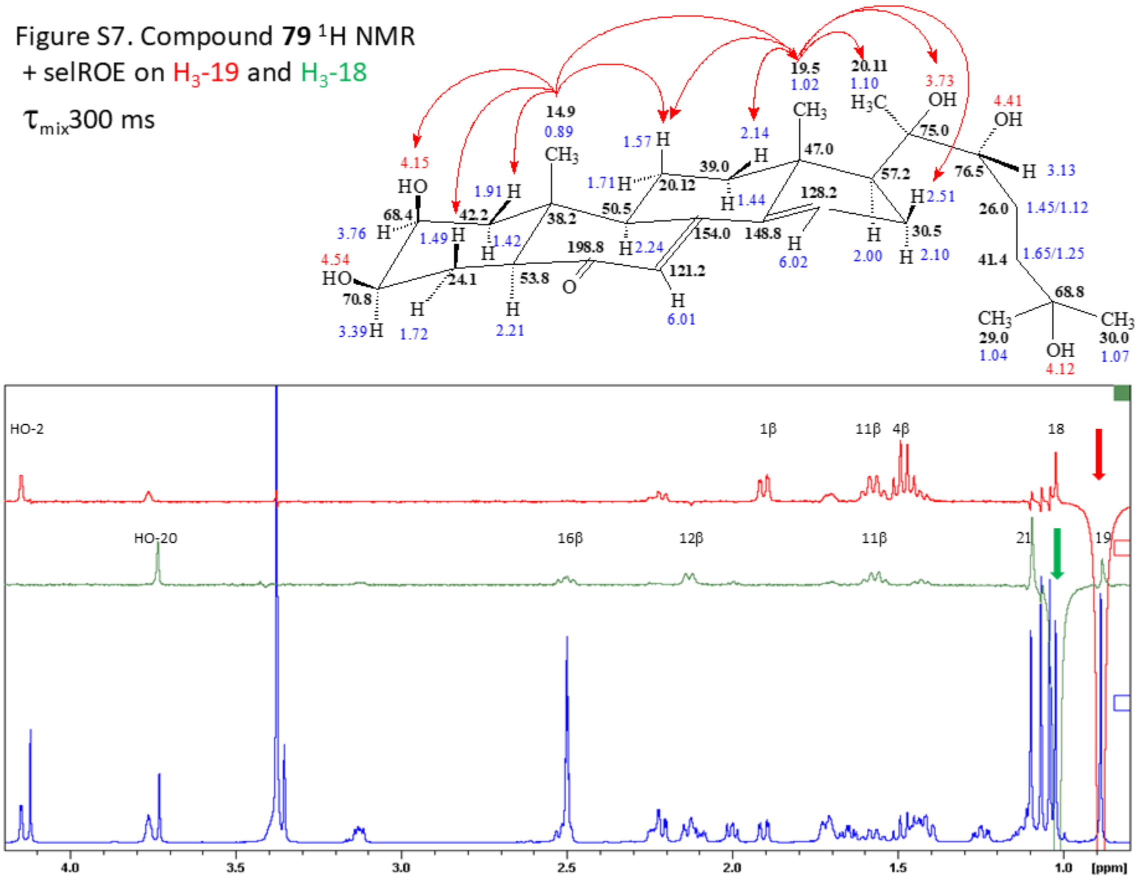

Figure S8. Compound **79** edHSQC
+ selROE on H_3-18

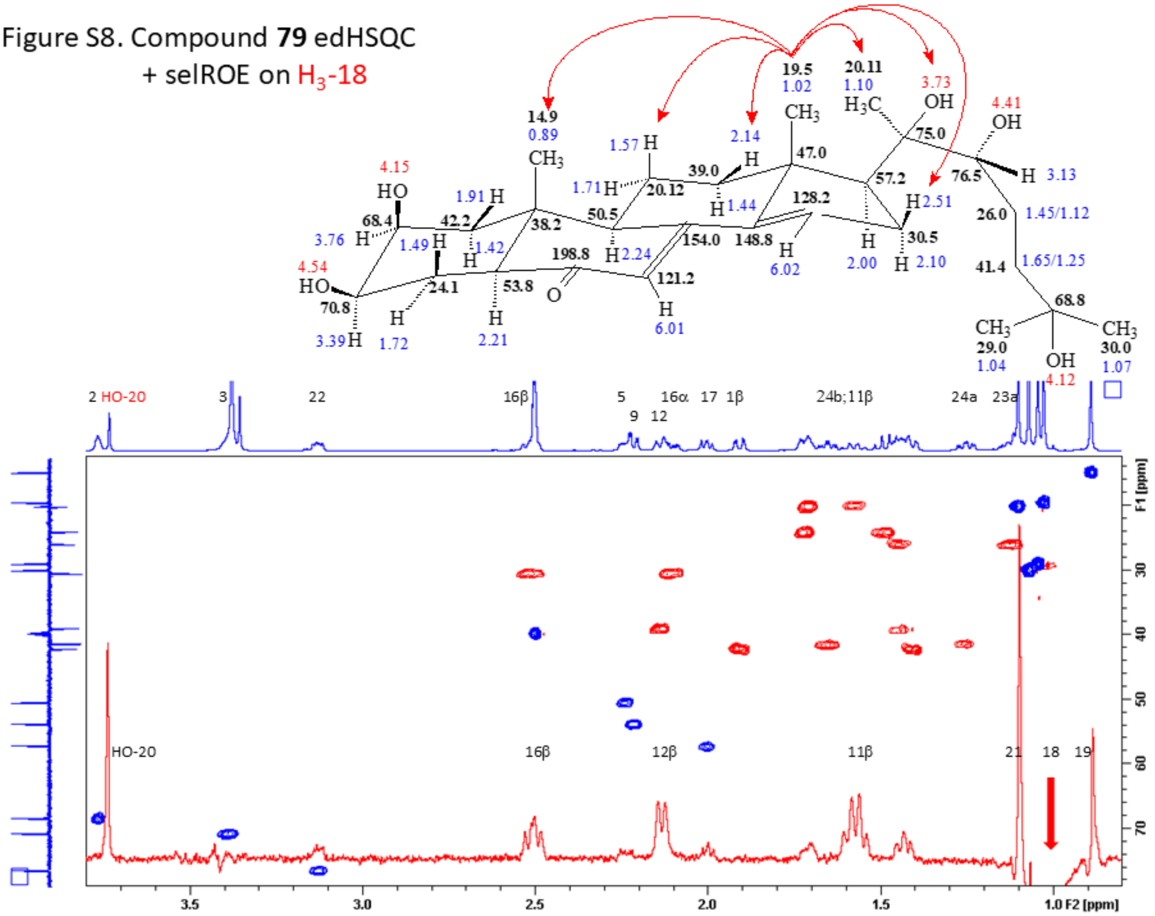

Figure S9. Compound **111** HRMS

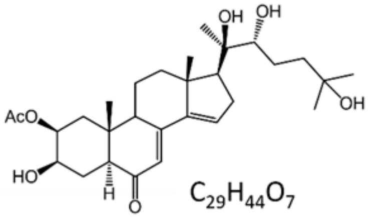

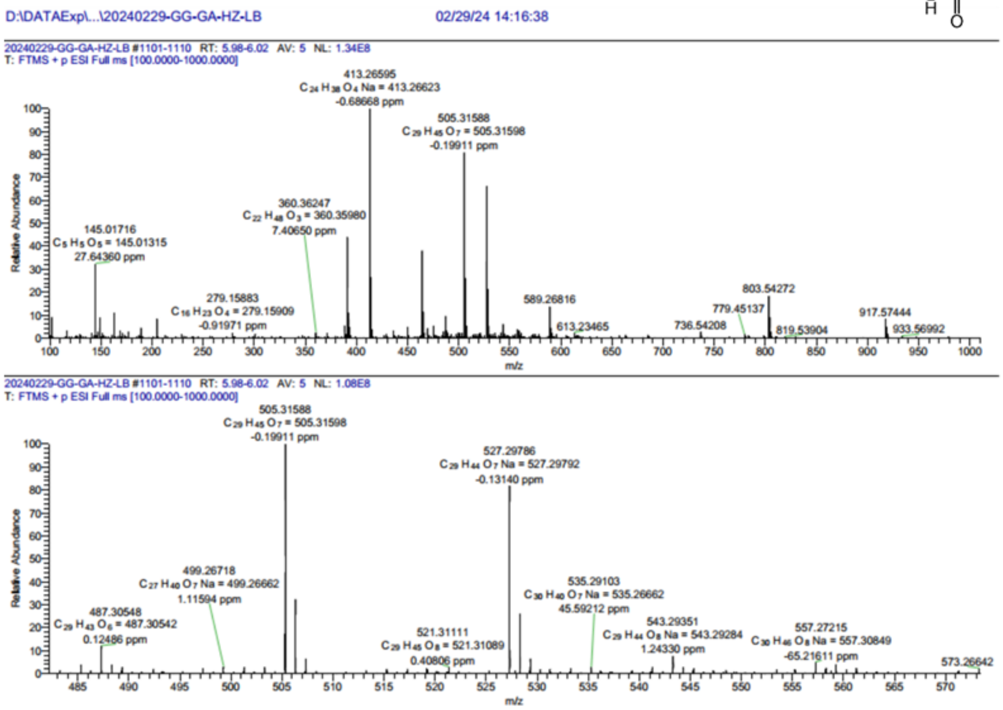

Figure S10. Compound **111** ^{1}H NMR

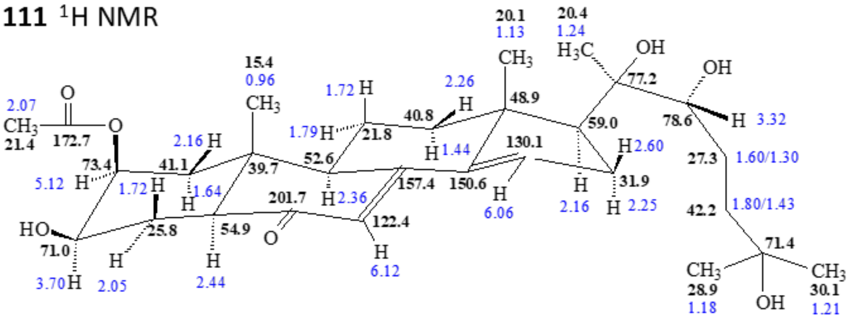

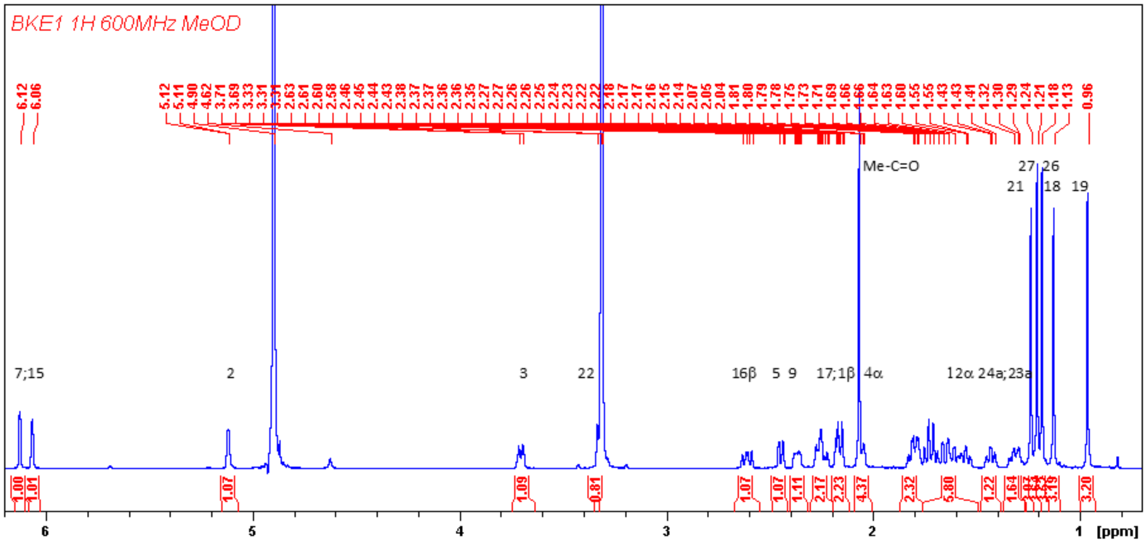

Figure S11. Compound **111**
^{13}C NMR+DEPT135

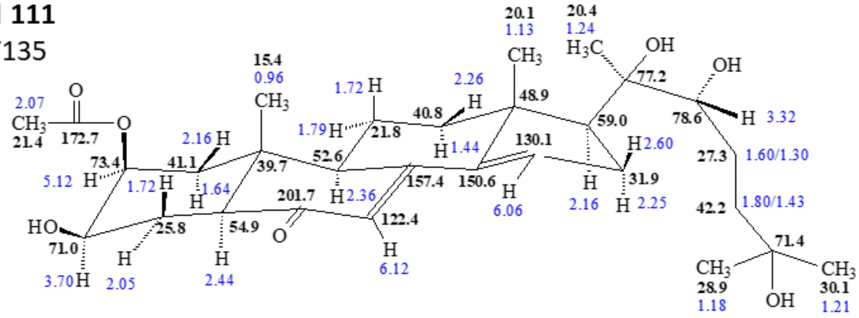

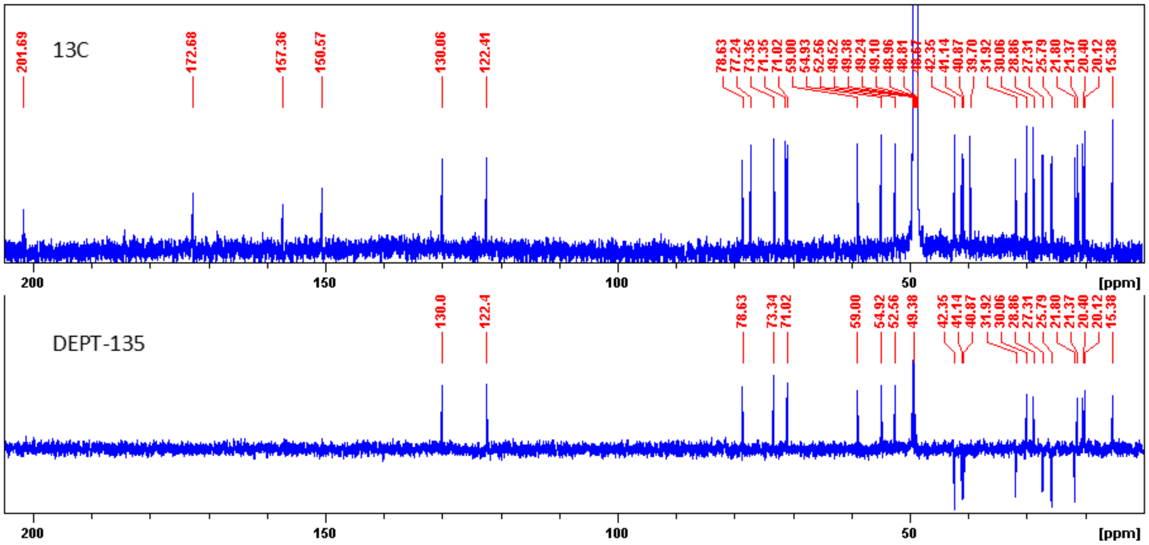

Figure S12. Compound **111** edHSQC

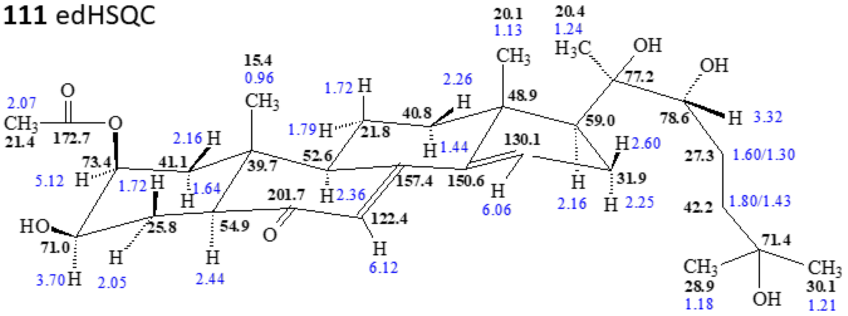

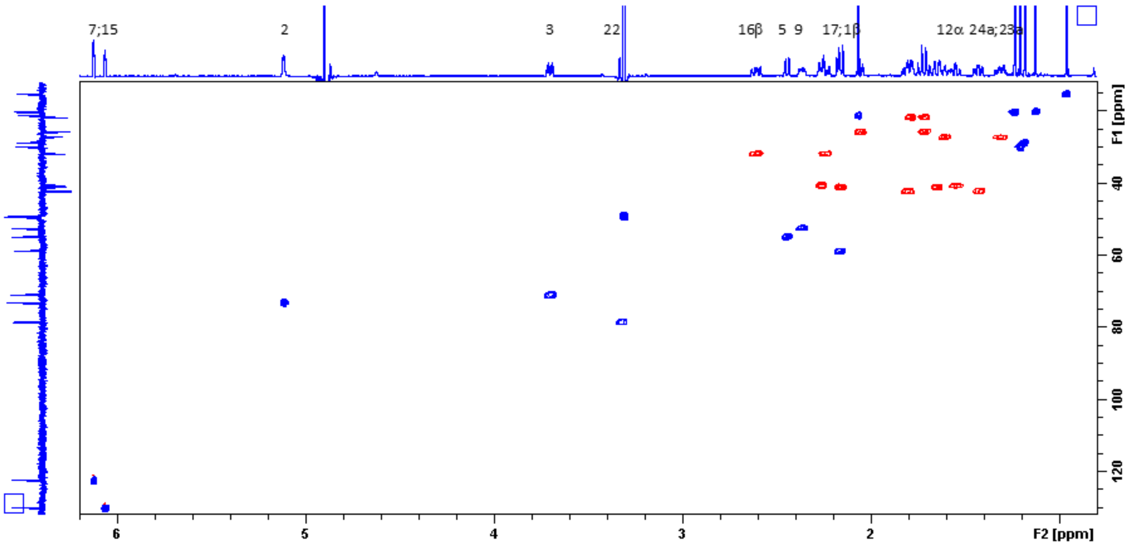

Figure S13. Compound **111** edHSQC

CH_2-section

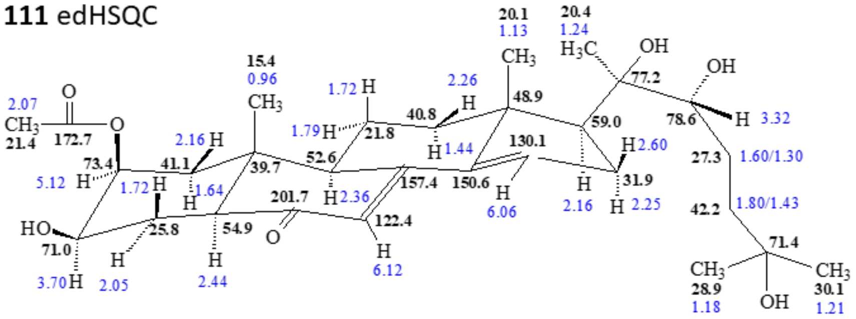

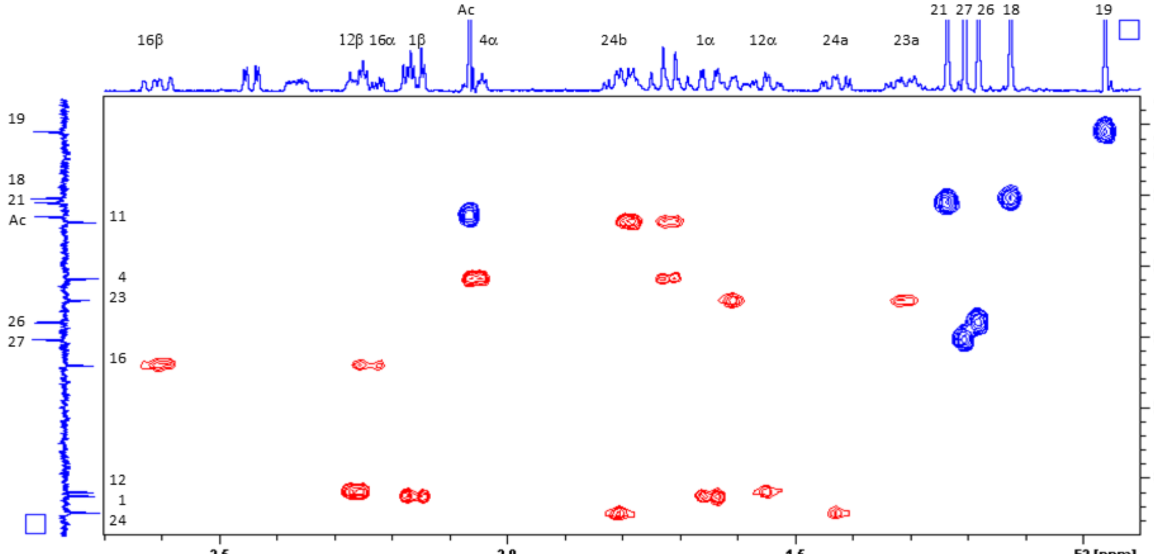

Figure S14. Compound **111** HMBC+Me-section

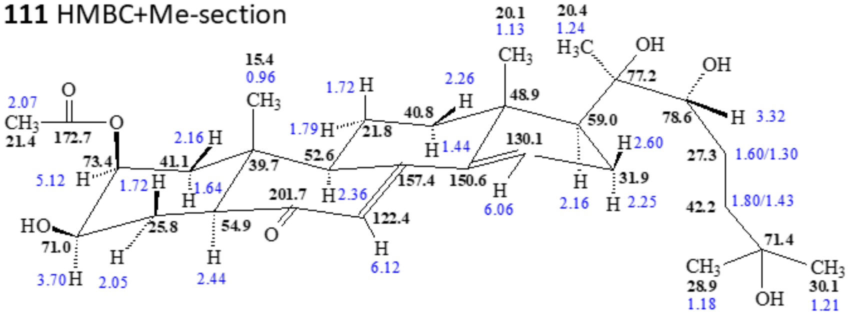

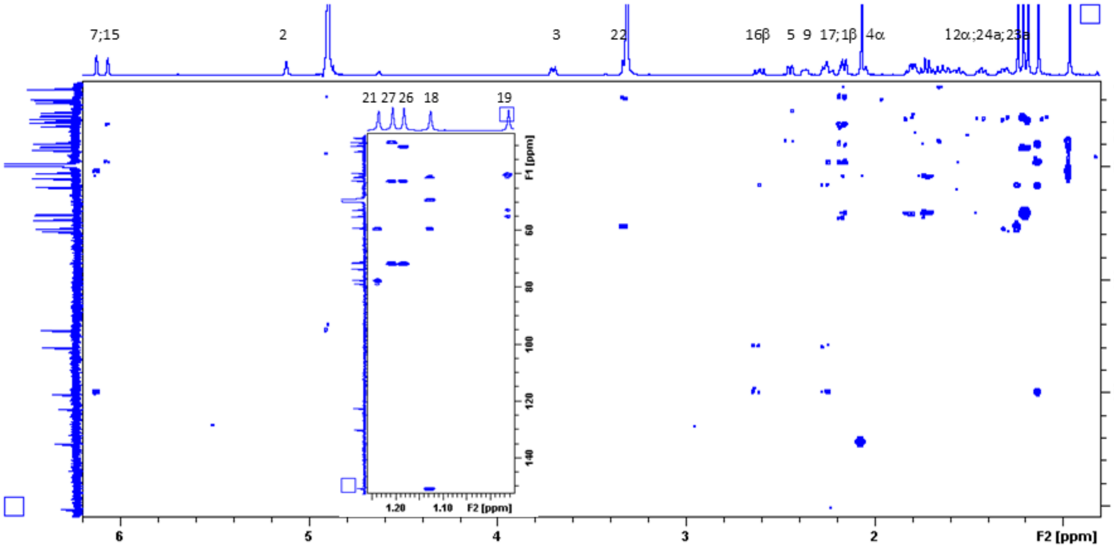

Figure S15. Compound **111** ^{1}H,^{1}H-COSY

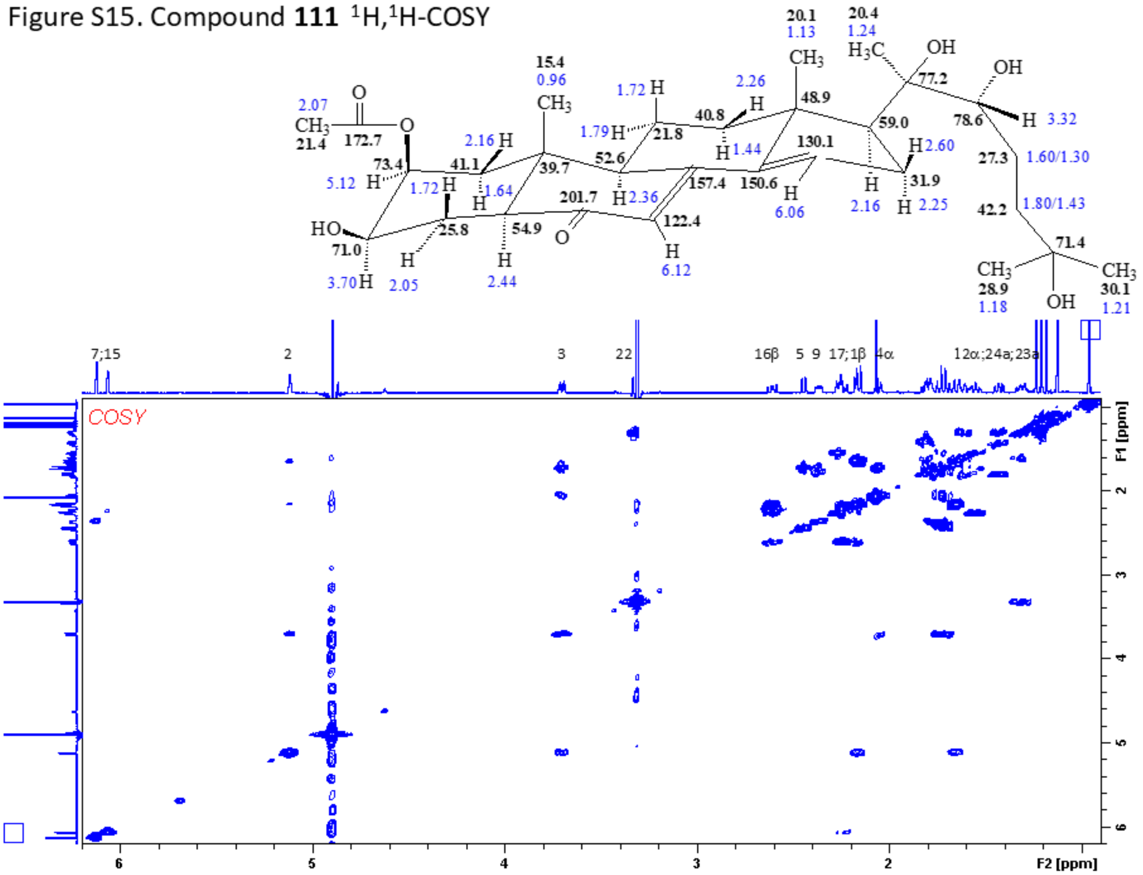

Figure S16 Compound **111** ^{1}H NMR
+ selROE on H_3-19 + selTOCSY on
H-15 and H-3

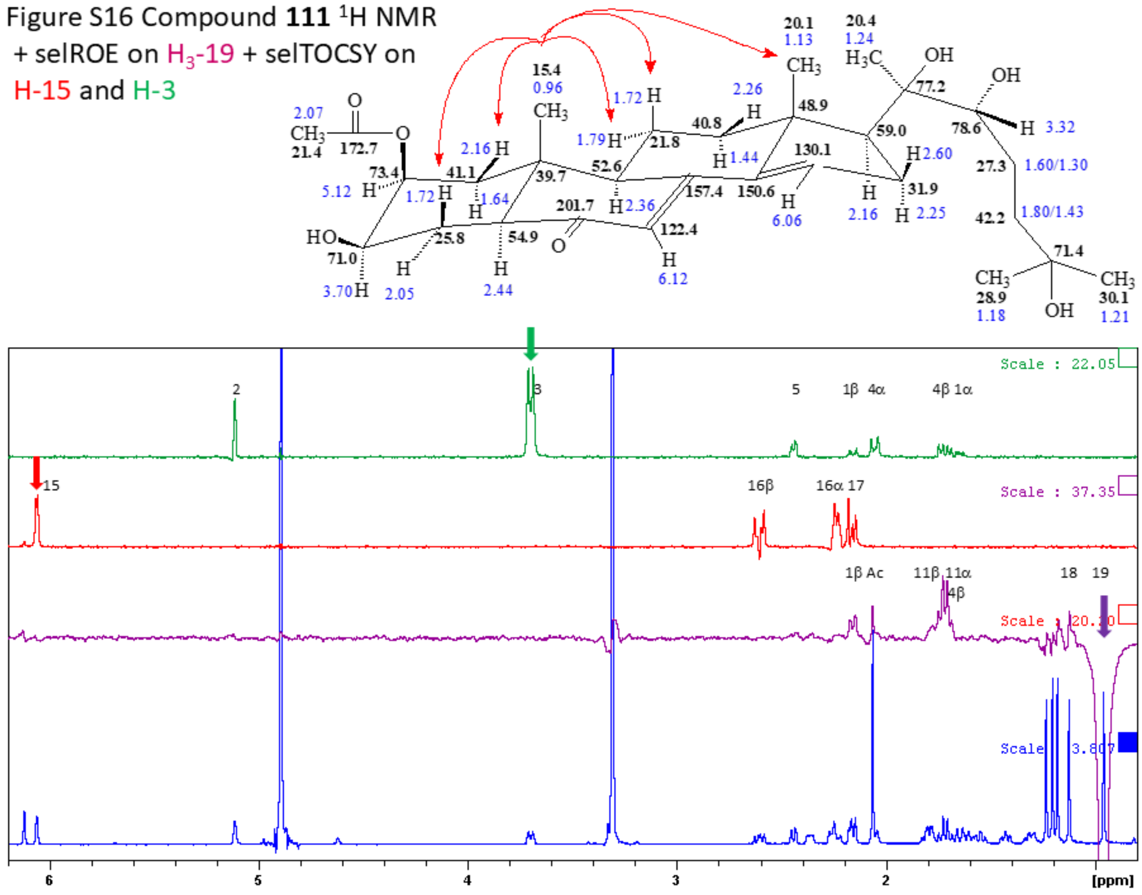

Figure S17. Compound **131** HRMS

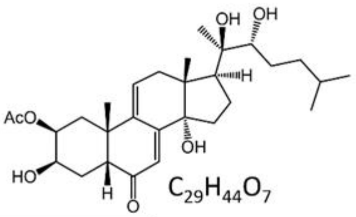

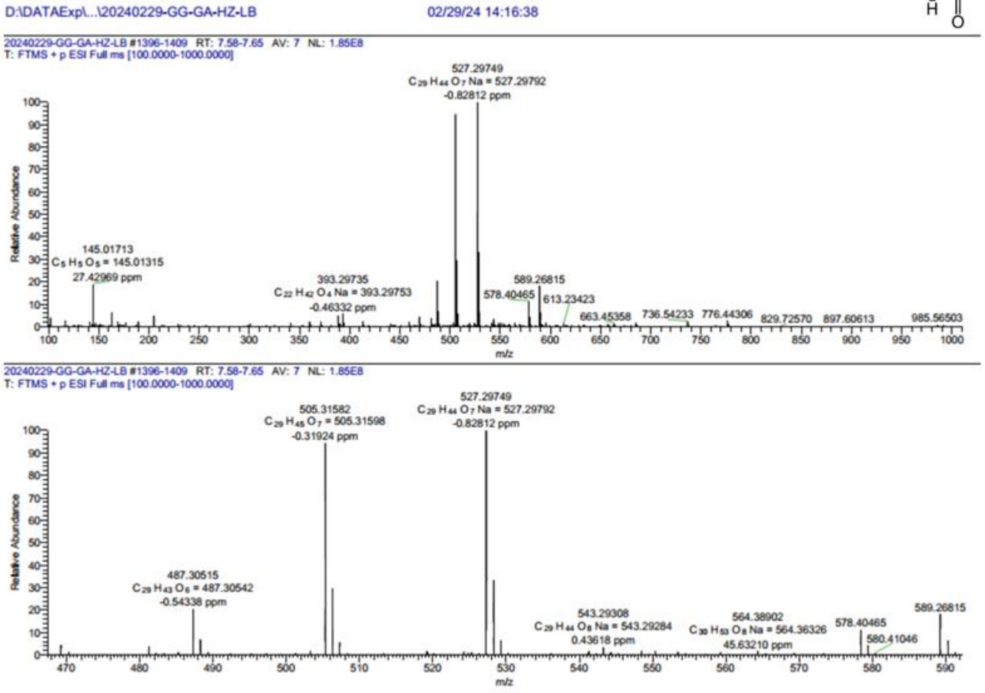

Figure S18. Compound **131** ^{1}H NMR

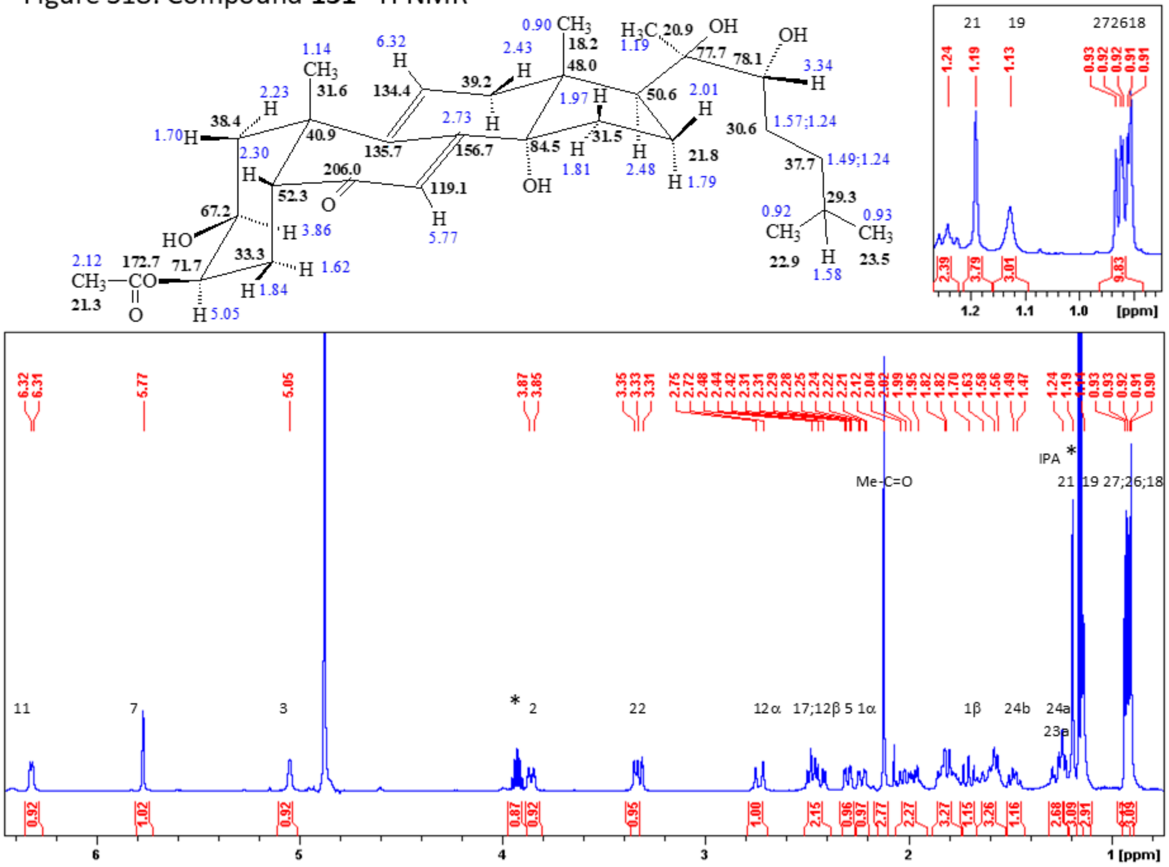

Figure S19. Compound **131** ^{13}C DEPTQ

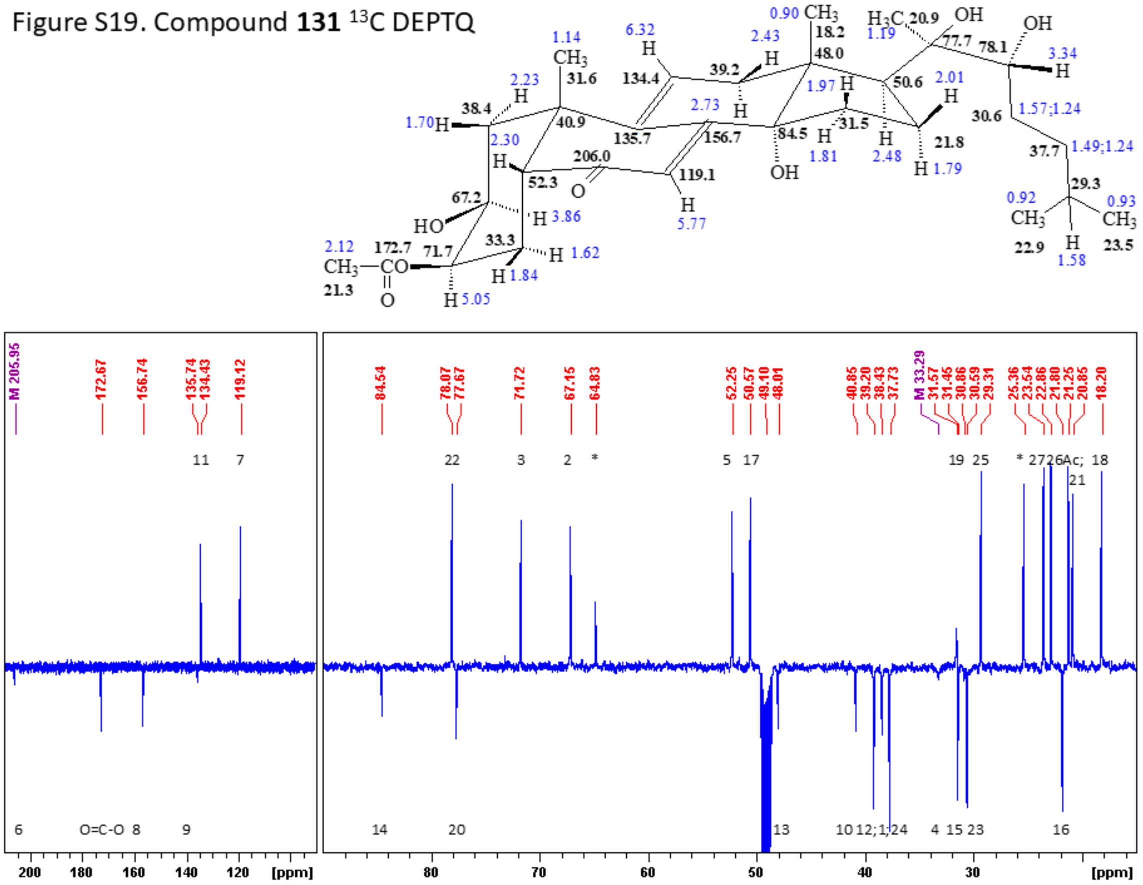

Figure S20. Compound **131** HSQC

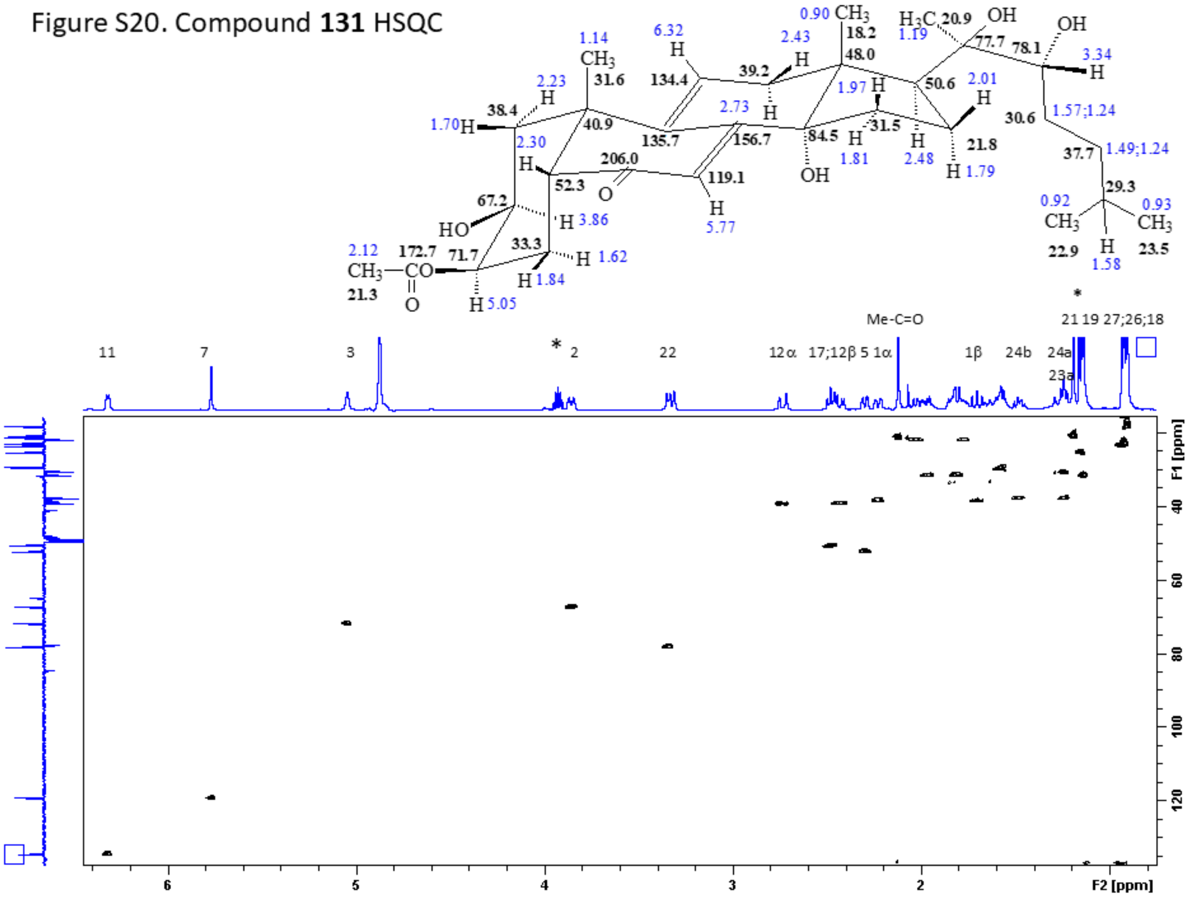

Figure S21. Compound **131** edHSQC

CH_2-section

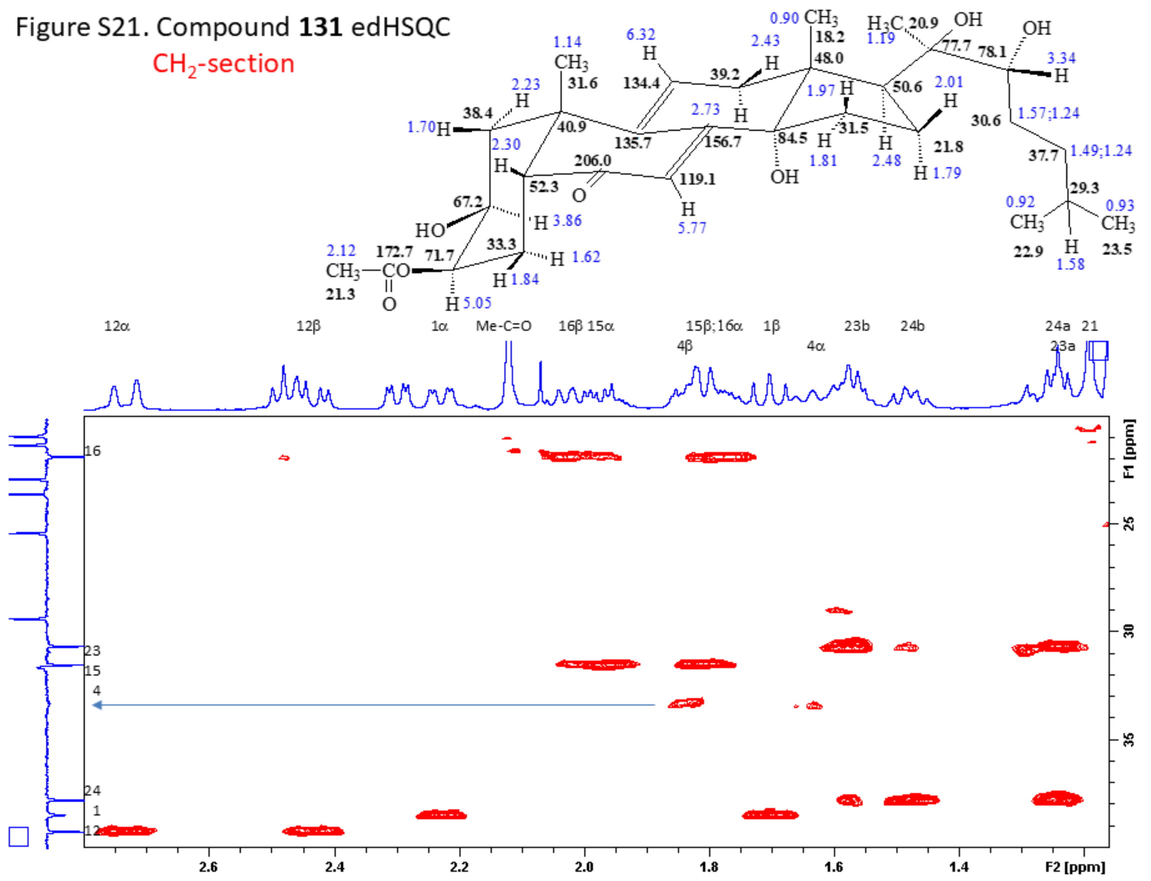

Figure S22. Compound **131** HMBC + Me-section

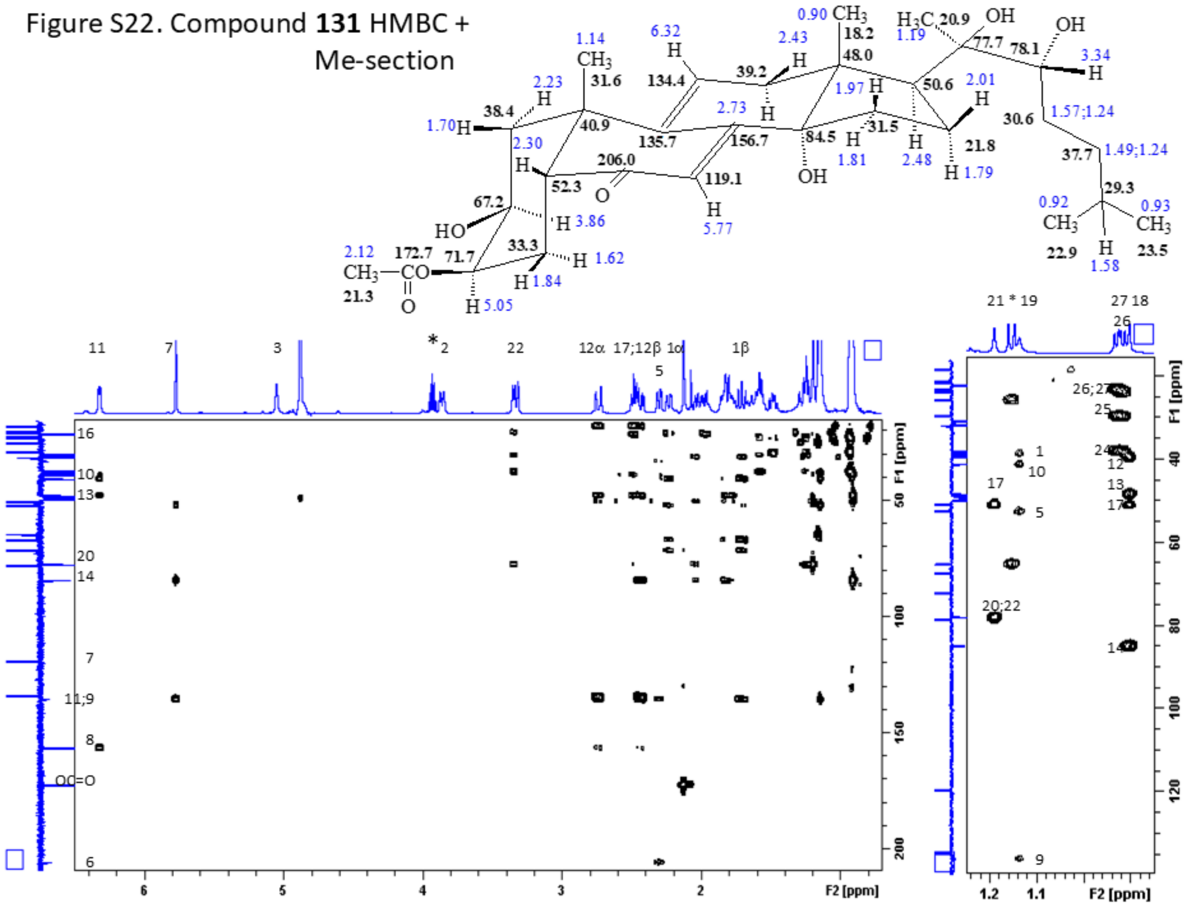

Figure S23. Compound **131** ^{1}H NMR + selROE on H_3-18(26;27); H_3-19; H_3-21

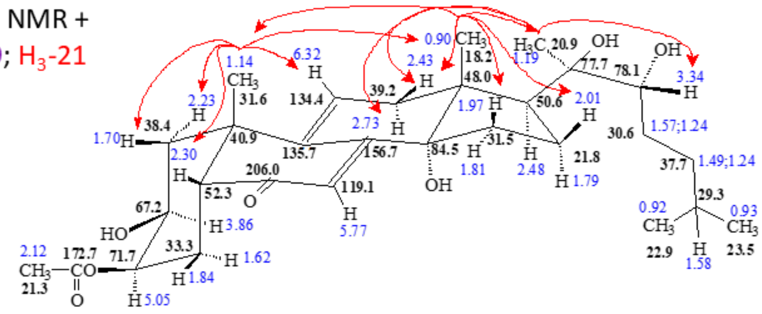

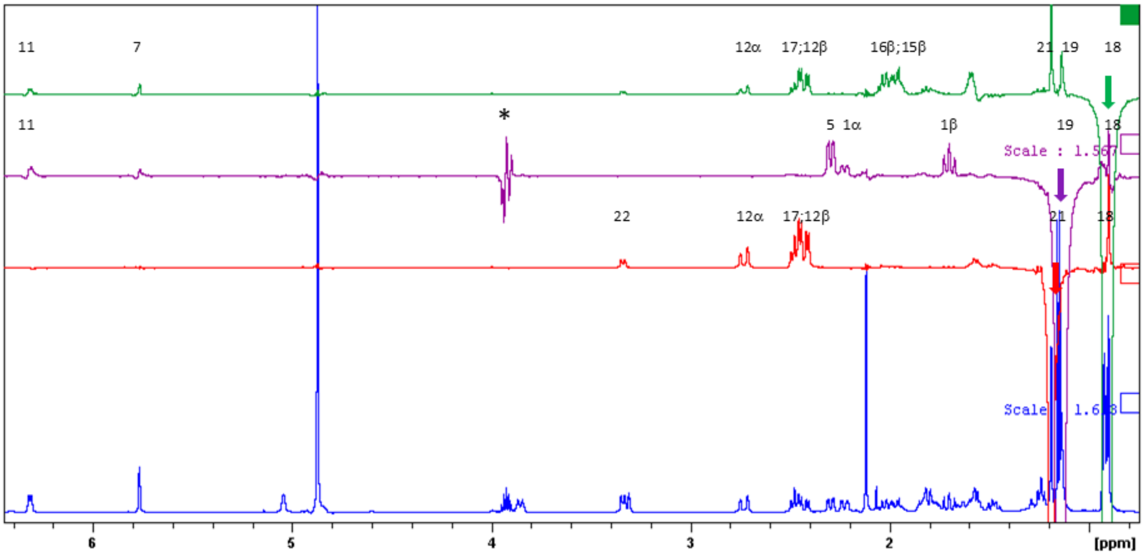

Figure S24. Compound **131** ^{1}H NMR + selTOCSY on H-2 and H-11

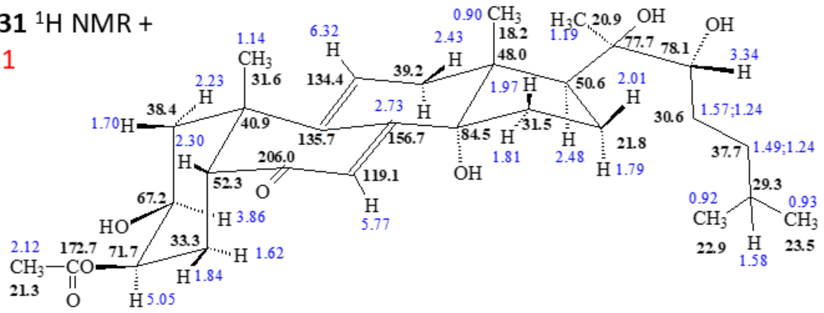

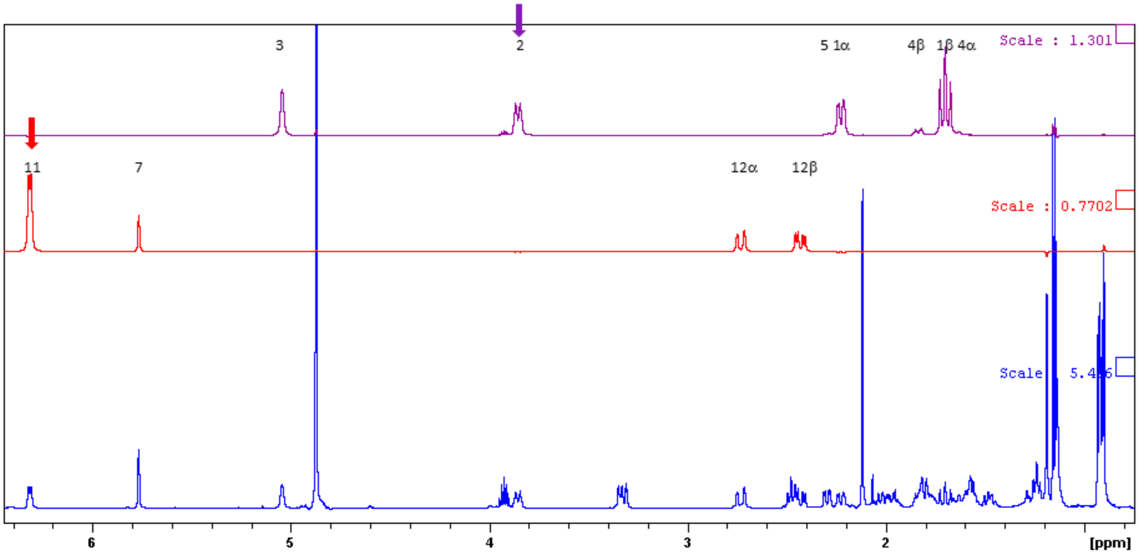

Figure S25. Compound **136** HRMS

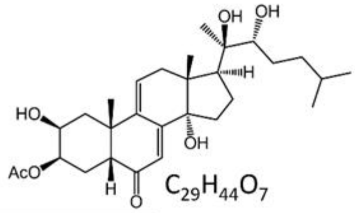

$C_{29}H_{44}O_7$

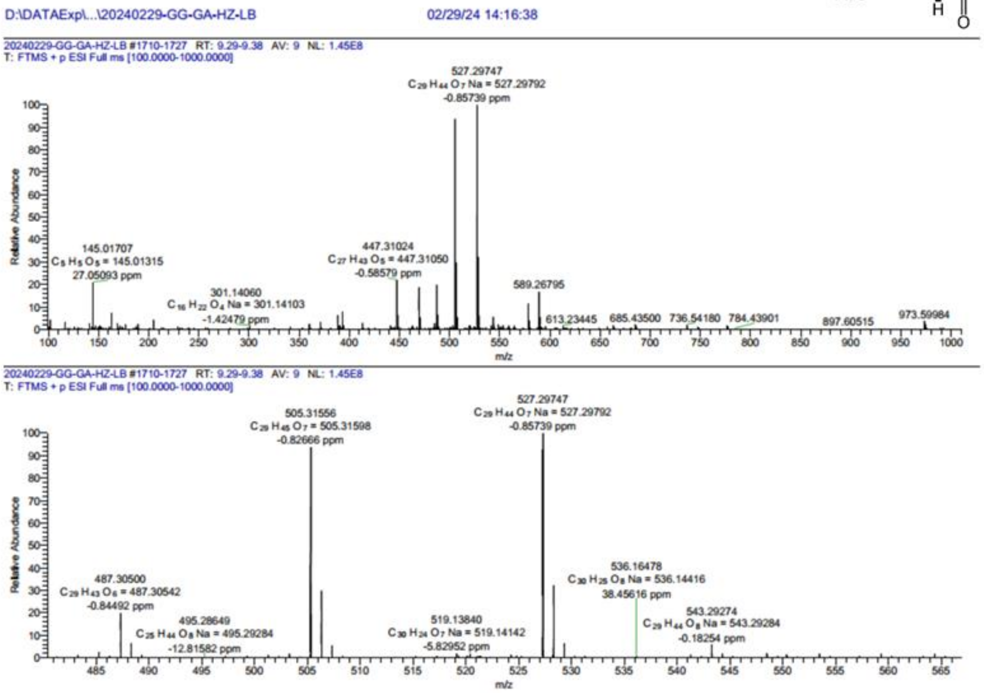

Figure S26. Compound **136** ^{1}H NMR

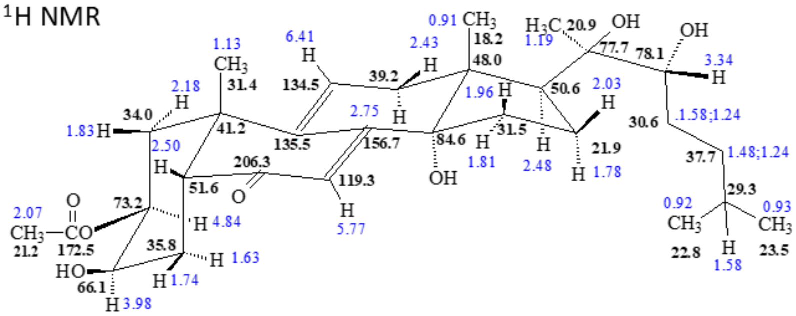

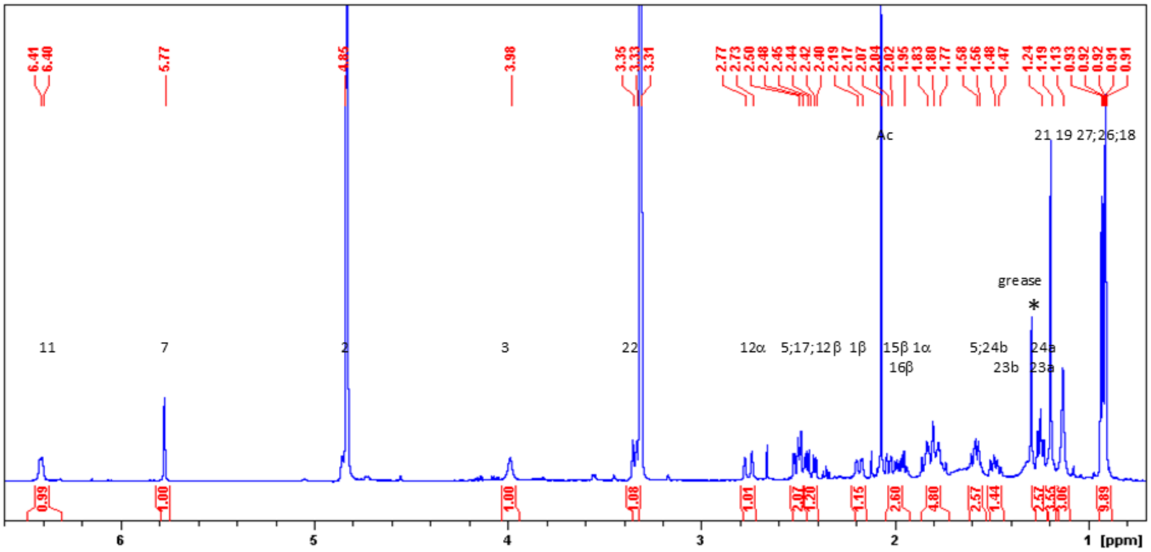

Figure S27. Compound **136** ^{13}C APT

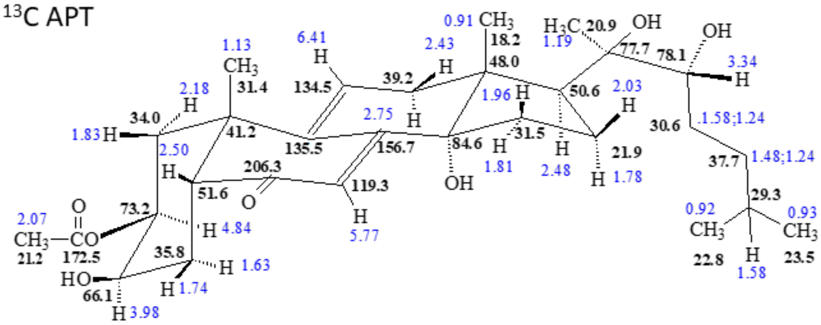

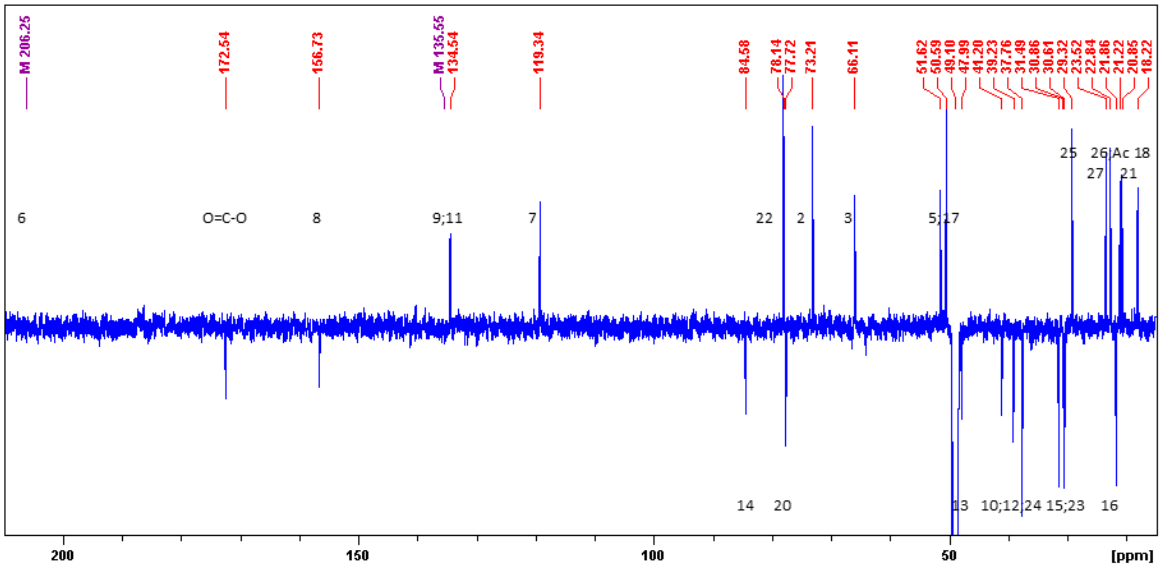

Figure S28. Compound **136** edHSQC

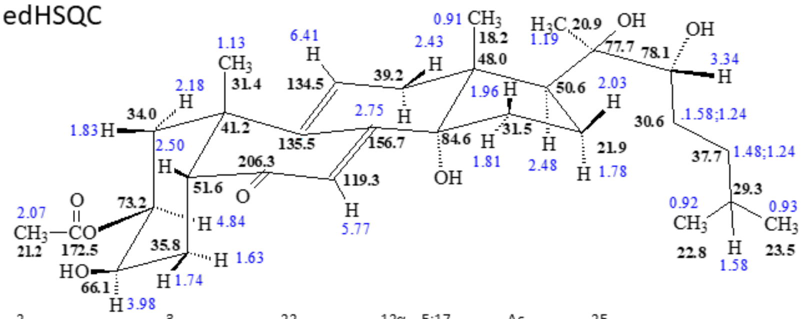

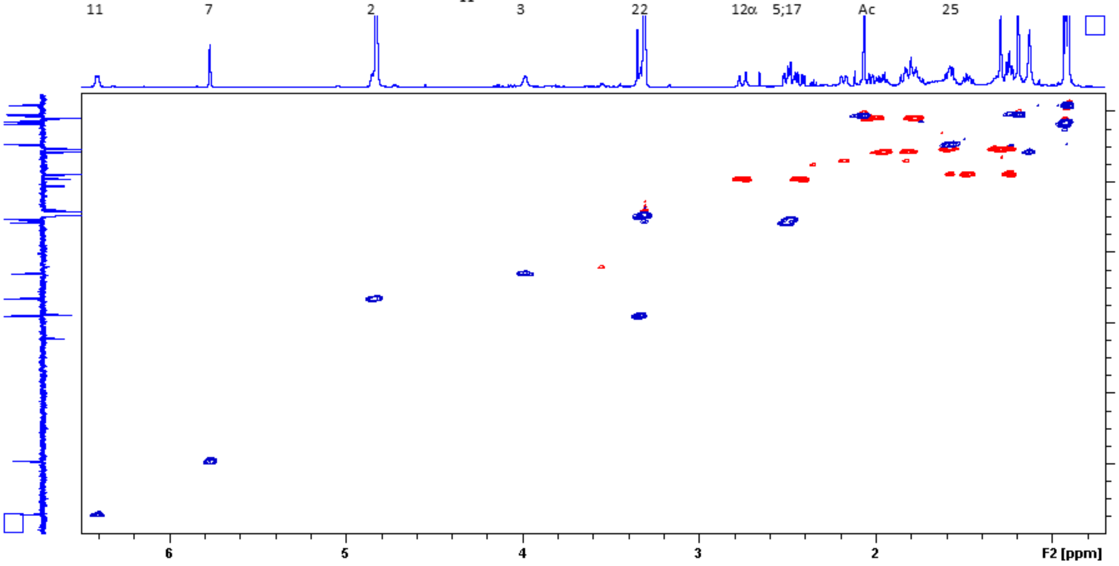

Figure S29. Compound **136** ^{1}H NMR selTOCSY on H-2, H-3

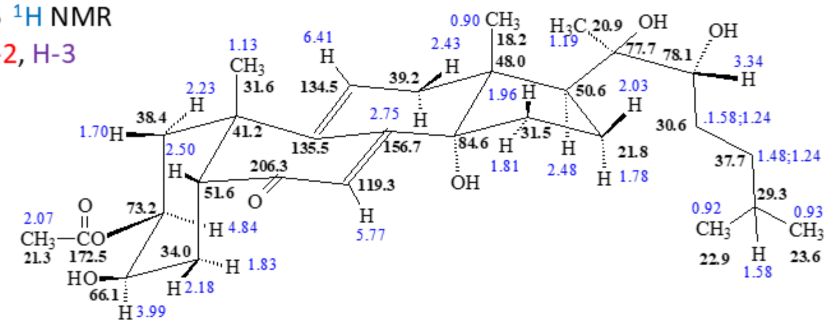

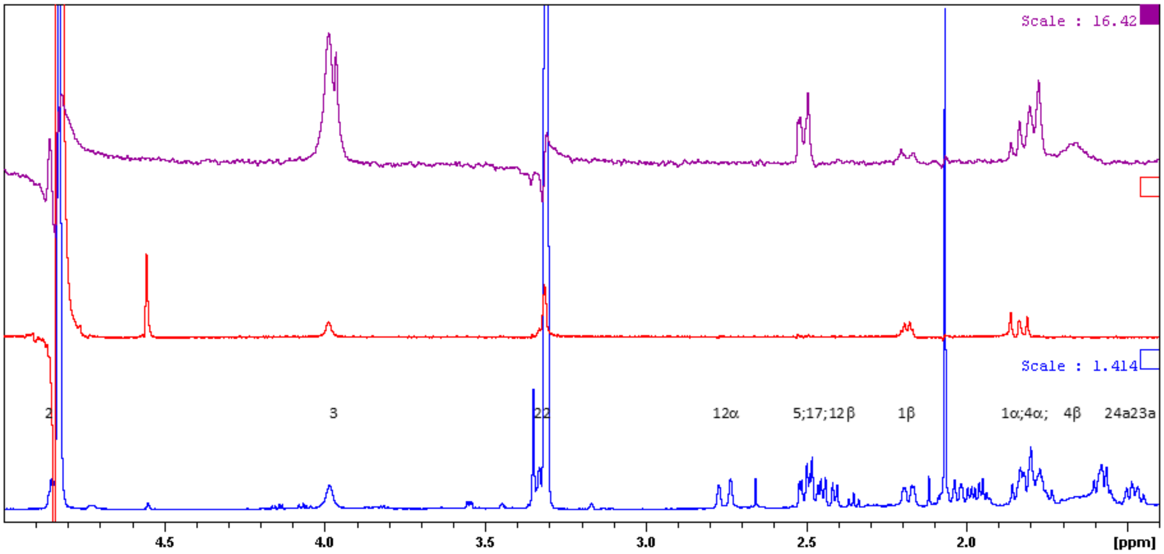

Figure S30. Compound **136** edHSQC CH_2-section + selTOCSY on H-2

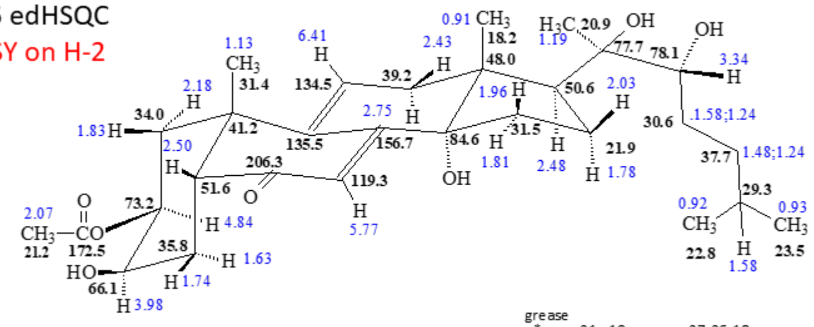

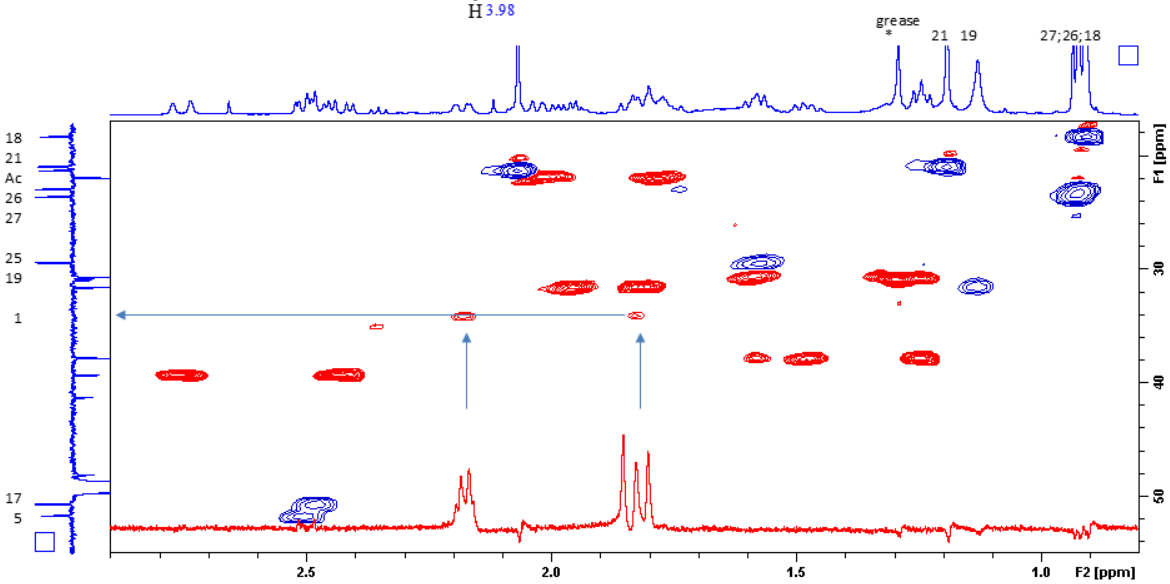

Figure S31. Compound **136** edHSQC
CH_2-section + selTOCSY on H-2; H-3

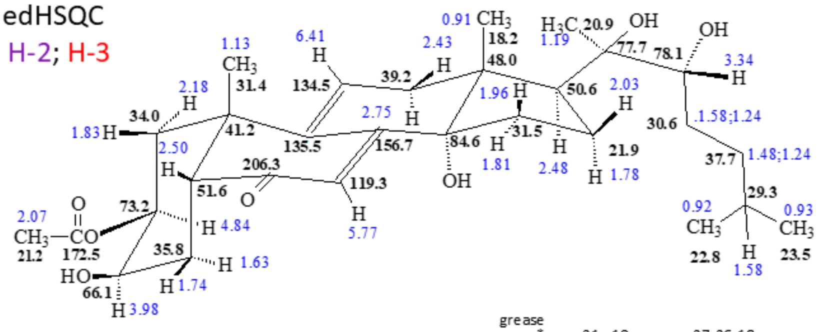

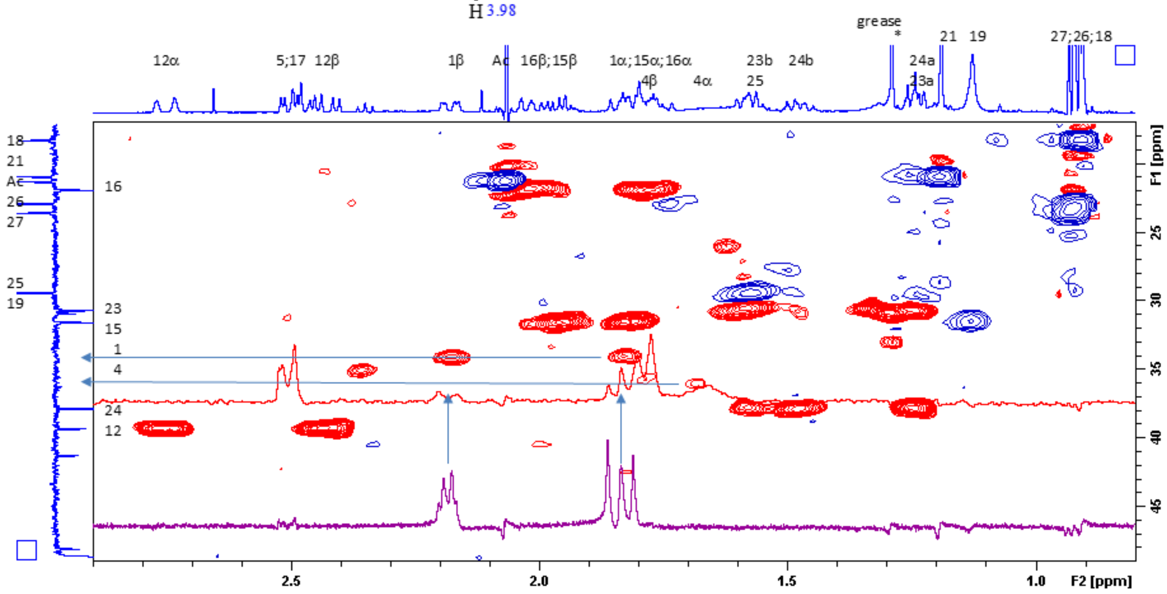

Figure S32. Compound **136** HMBC +
Me-section

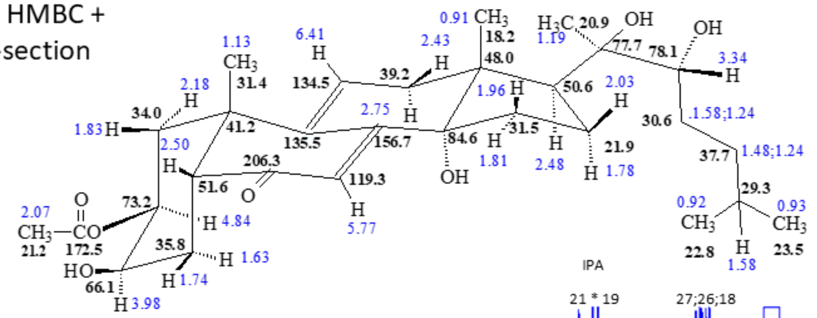

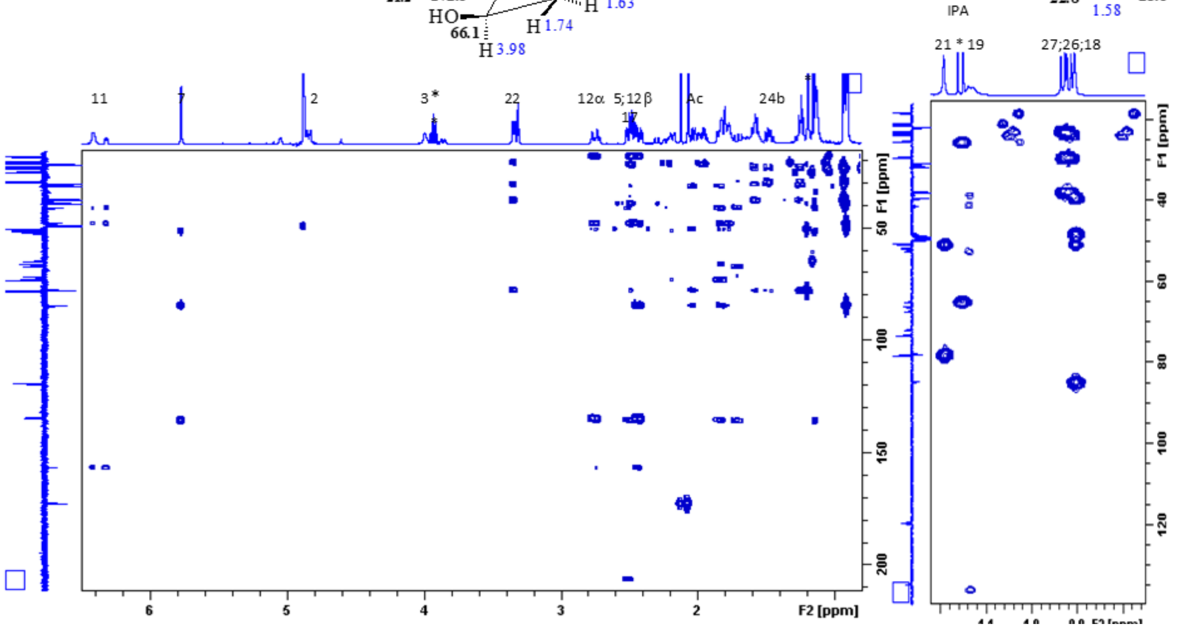

Figure S33. Compound **135** HRMS

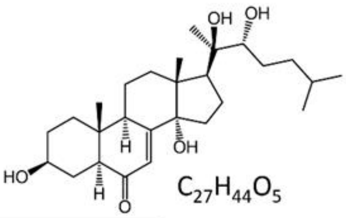

$C_{27}H_{44}O_5$

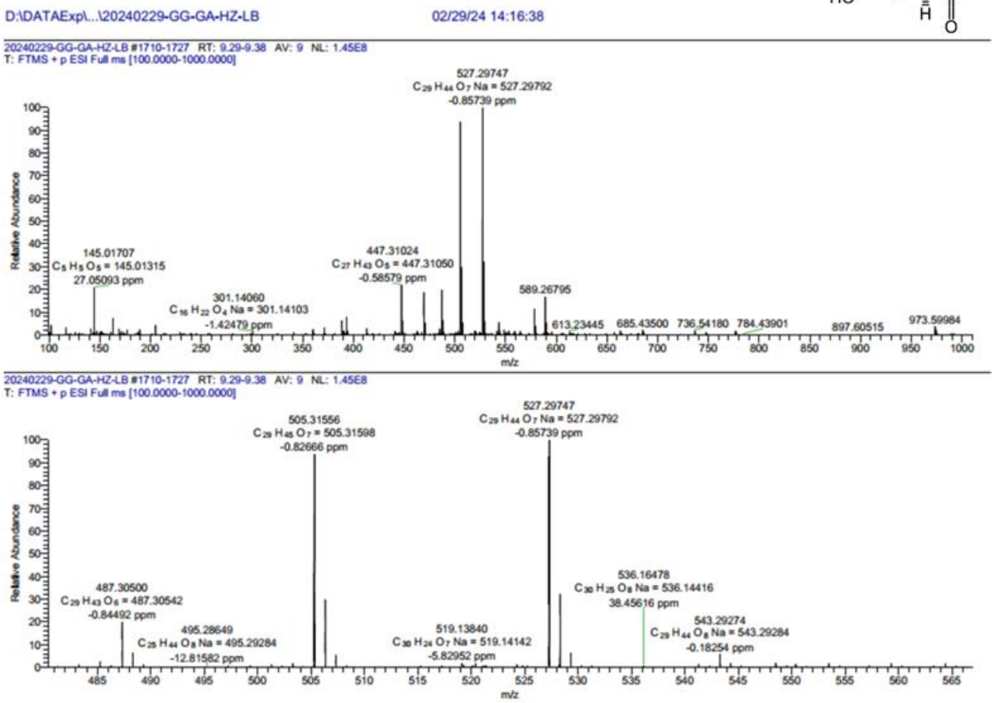

Figure S34. Compound **135** ^{1}H NMR

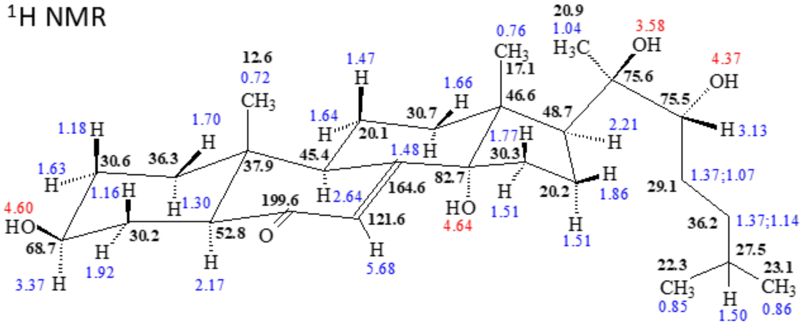

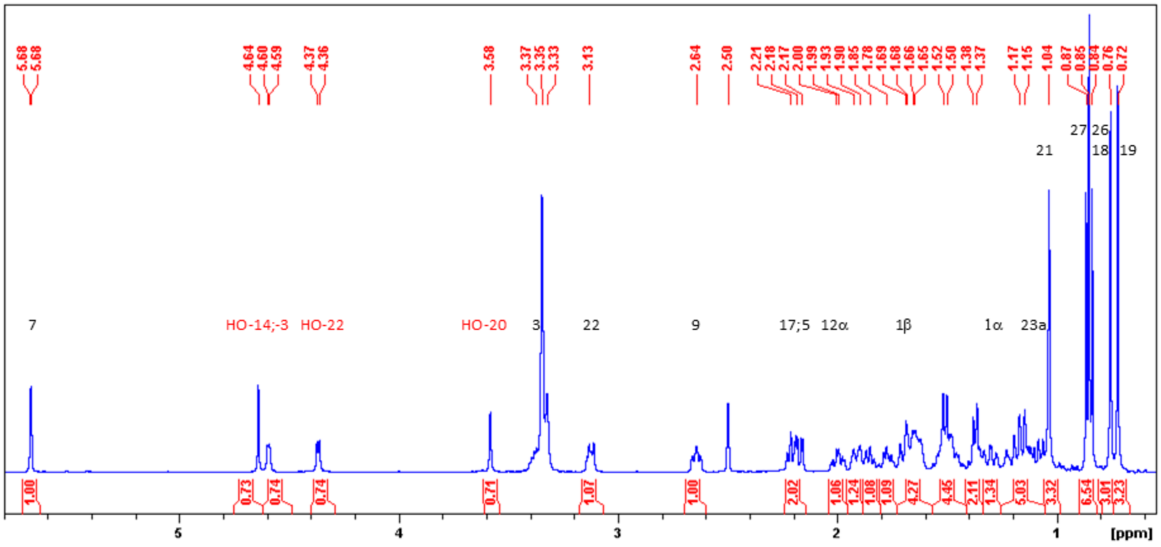

Figure S35. Compound **135** ^{13}C DEPTQ

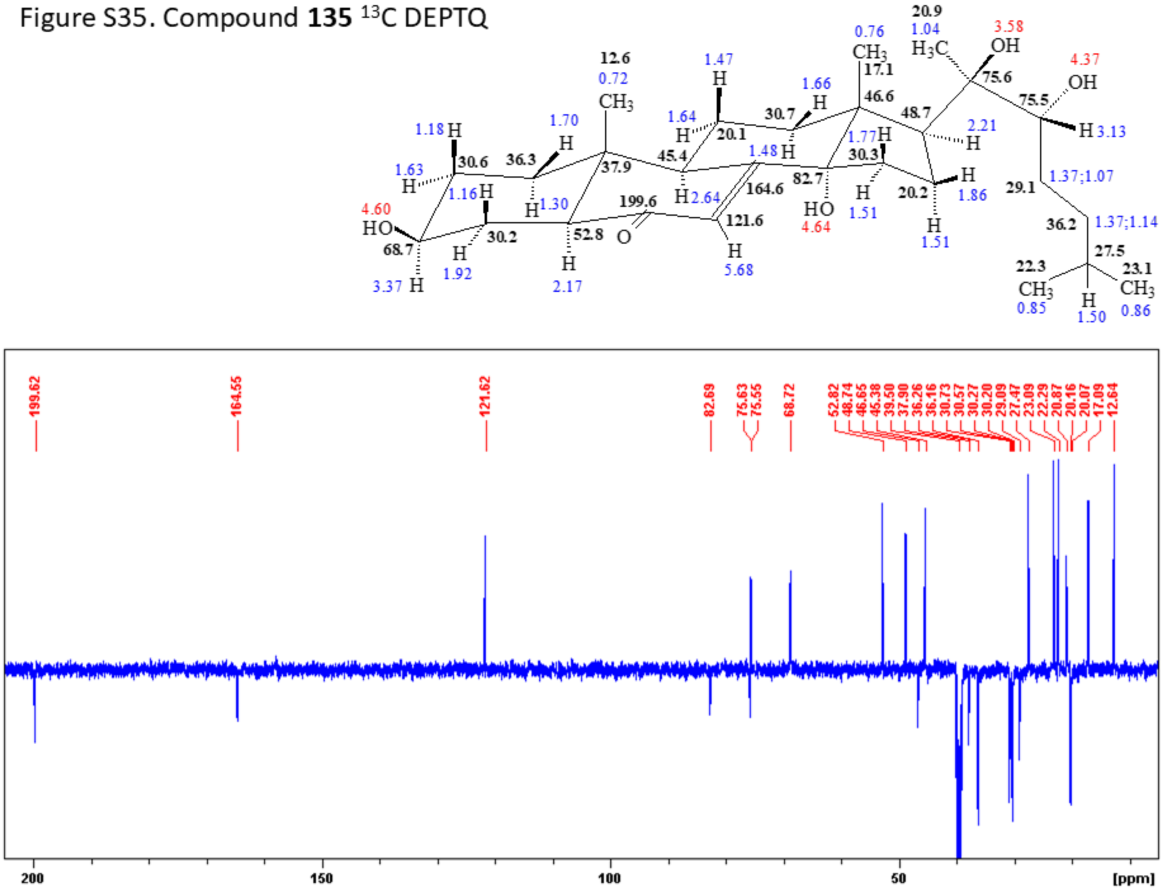

Figure S36. Compound **135** HSQC

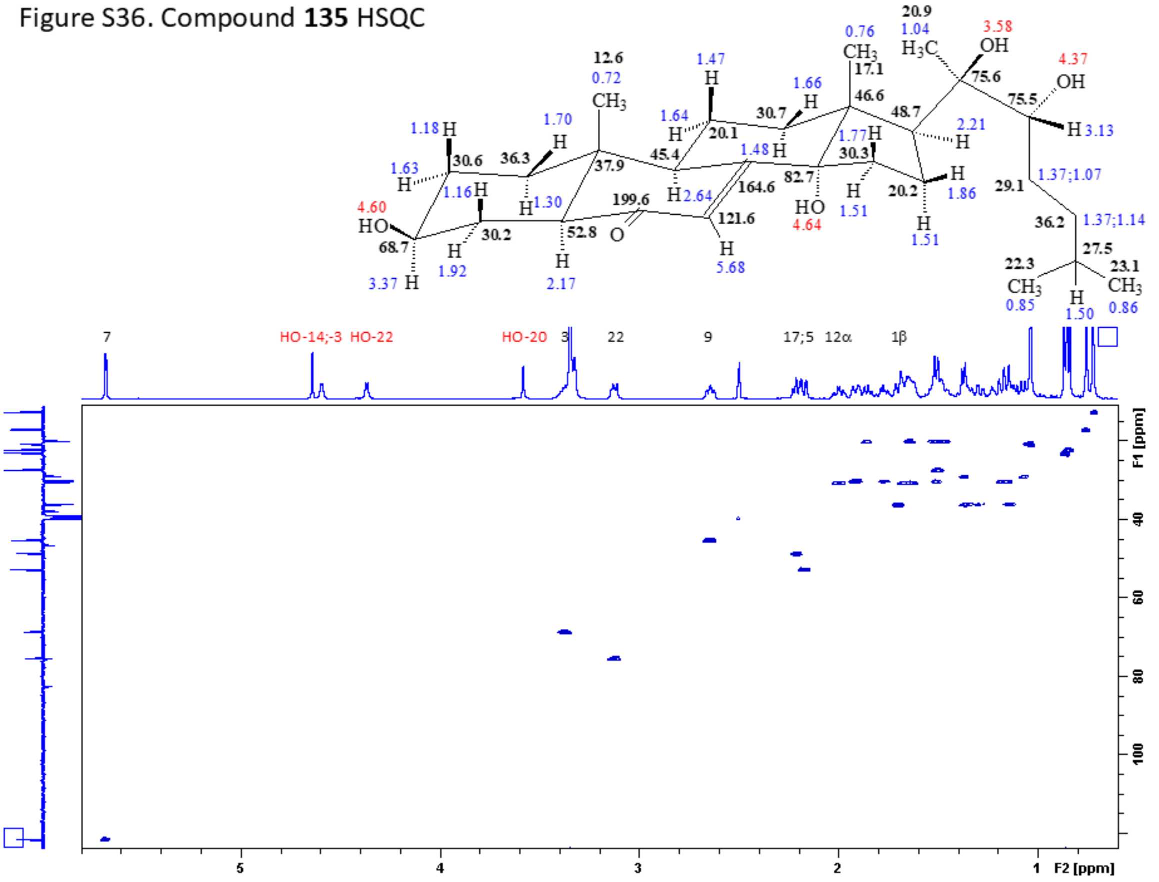

Figure S37. Compound **135** edHSQC
CH_3- and CH_2-section

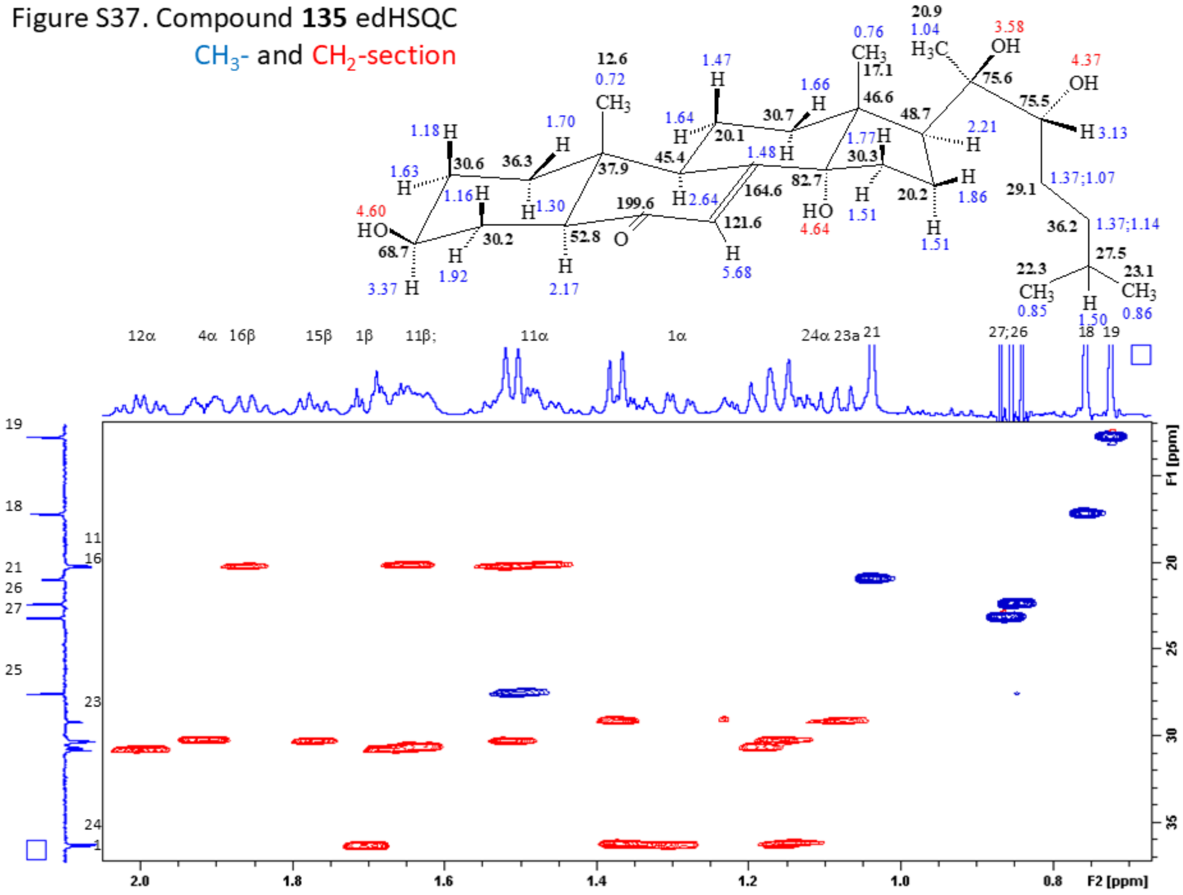

Figure S38. Compound **135** HMBC
+ Me-section

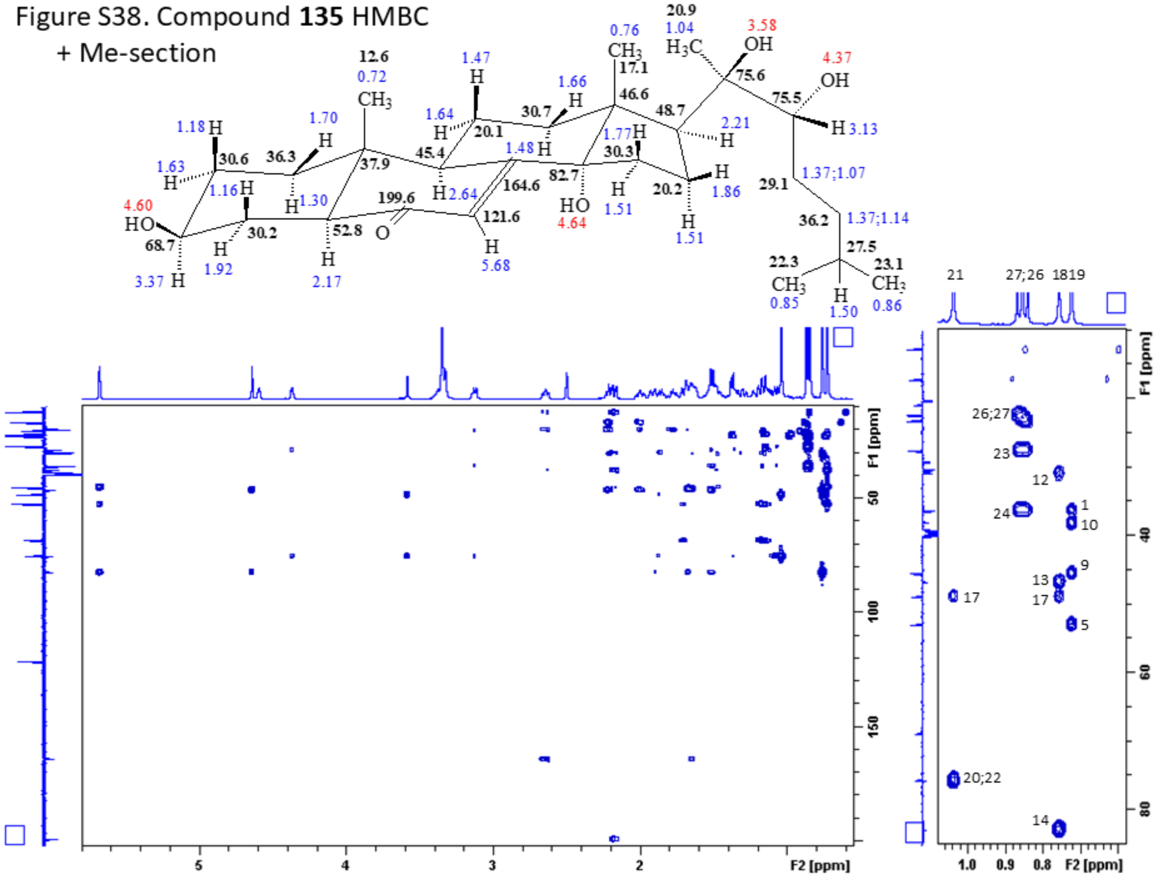

Figure S39. Compound **135** ^{1}H NMR +
+ selROE and selTOCSY on Hα-5

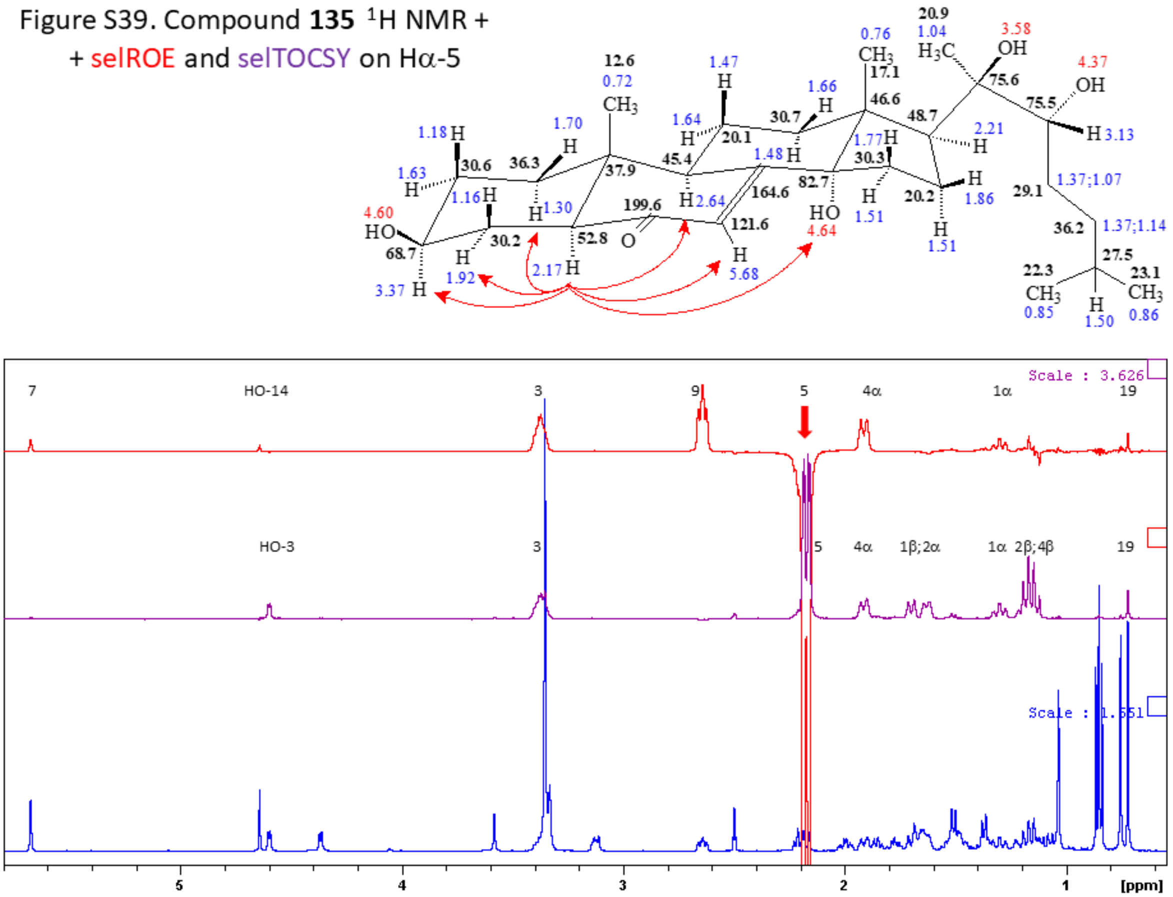

Figure S40. Compound **135** ^{1}H NMR
+ selROE on H_3-19 and H_3-18

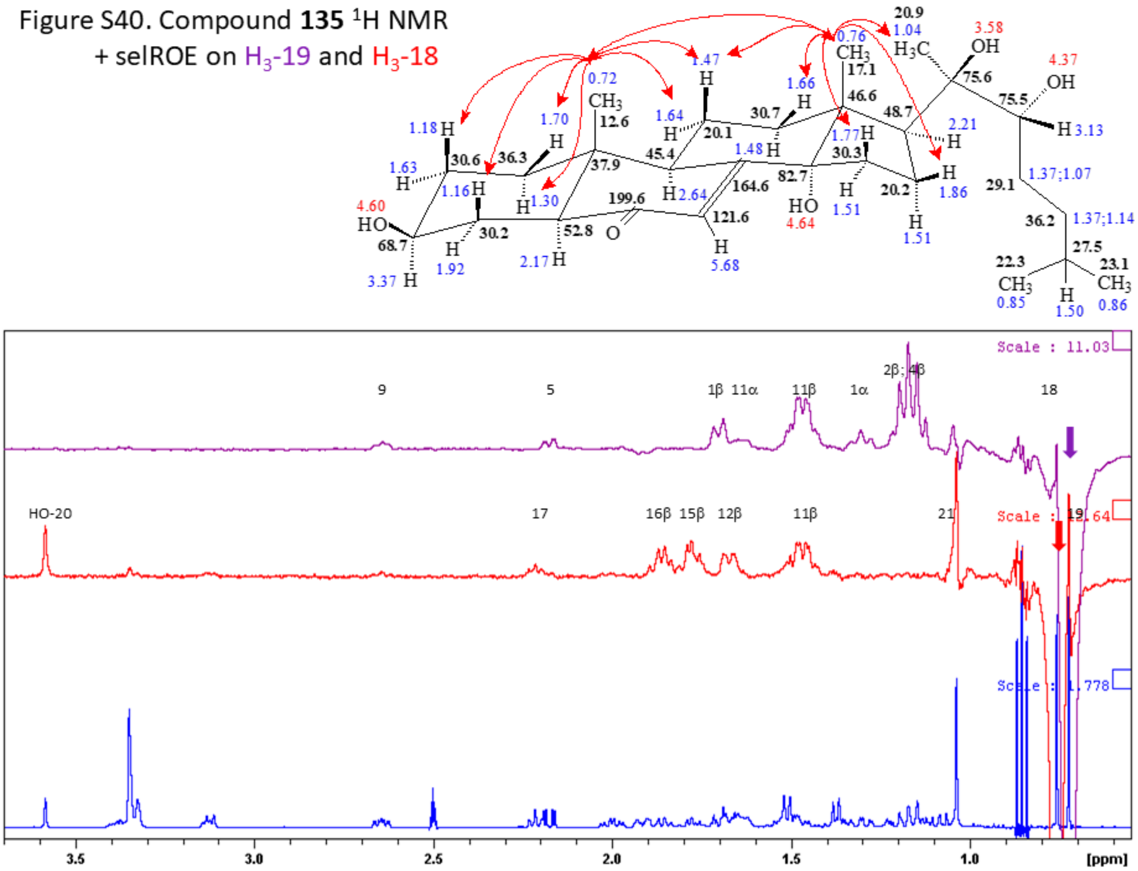

Supplement: Supplementary file 1 — ao4c10908_si_001.pdf [file ao4c10908_si_001.pdf]
